# HDAC class I inhibitor domatinostat sensitizes pancreatic cancer to chemotherapy by targeting cancer stem cell compartment via FOXM1 modulation

**DOI:** 10.1186/s13046-022-02295-4

**Published:** 2022-03-03

**Authors:** Maria Serena Roca, Tania Moccia, Federica Iannelli, Cristina Testa, Carlo Vitagliano, Michele Minopoli, Rosa Camerlingo, Giulia De Riso, Rossella De Cecio, Francesca Bruzzese, Mariarosaria Conte, Lucia Altucci, Elena Di Gennaro, Antonio Avallone, Alessandra Leone, Alfredo Budillon

**Affiliations:** 1 Experimental Pharmacology Unit-Laboratory of Naples and Mercogliano (AV), Istituto Nazionale per lo Studio e la Cura dei Tumori “Fondazione G. Pascale” - IRCCS, Naples, Italy; 2 Neoplastic Progression Unit, Istituto Nazionale per lo Studio e la Cura dei Tumori “Fondazione G. Pascale” – IRCCS, Naples, Italy; 3 Cell Biology and Biotherapy Unit, Istituto Nazionale per lo Studio e la Cura dei Tumori “Fondazione G. Pascale” – IRCCS, Naples, Italy; 4 Department of molecular medicine and medical biotechnology, University of Naples “Federico II”, Naples, Italy; 5 Pathology Unit, Istituto Nazionale per lo Studio e la Cura dei Tumori “Fondazione G. Pascale” – IRCCS, Naples, Italy; 6 Animal Facility, Istituto Nazionale per lo Studio e la Cura dei Tumori “Fondazione G. Pascale” – IRCCS, Naples, Italy; 7 Department of Precision Medicine, Università degli Studi della Campania “Luigi Vanvitelli”, Naples, Italy; 8 BIOGEM, (AV), Naples, Italy; 9 Experimental Clinical Abdominal Oncology; Istituto Nazionale per lo Studio e la Cura dei Tumori “Fondazione G. Pascale” – IRCCS, Naples, Italy

**Keywords:** HDAC inhibitors, Domatinostat, Drug resistance, FOXM1, Cancer stem cells, Stress adaptation, Pancreatic cancer

## Abstract

**Background:**

Pancreatic ductal adenocarcinoma (PDAC) represents an unmet clinical need due to the very poor prognosis and the lack of effective therapy. Here we investigated the potential of domatinostat (4SC-202), a new class I histone deacetylase (HDAC) inhibitor, currently in clinical development, to sensitize PDAC to first line standard gemcitabine (G)/taxol (T) doublet chemotherapy treatment.

**Methods:**

Synergistic anti-tumor effect of the combined treatment was assessed in PANC1, ASPC1 and PANC28 PDAC cell lines in vitro as well as on tumor spheroids and microtissues, by evaluating combination index (CI), apoptosis, clonogenic capability. The data were confirmed in vivo xenograft models of PANC28 and PANC1 cells in athymic mice. Cancer stem cells (CSC) targeting was studied by mRNA and protein expression of CSC markers, by limiting dilution assay, and by flow cytometric and immunofluorescent evaluation of CSC mitochondrial and cellular oxidative stress. Mechanistic role of forkhead box M1 (FOXM1) and downstream targets was evaluated in FOXM1-overexpressing PDAC cells.

**Results:**

We showed that domatinostat sensitized in vitro and in vivo models of PDAC to chemotherapeutics commonly used in PDAC patients management and particularly to GT doublet, by targeting CSC compartment through the induction of mitochondrial and cellular oxidative stress. Mechanistically, we showed that domatinostat hampers the expression and function of FOXM1, a transcription factor playing a crucial role in stemness, oxidative stress modulation and DNA repair. Domatinostat reduced FOXM1 protein levels by downregulating mRNA expression and inducing proteasome-mediated protein degradation thus preventing nuclear translocation correlated with a reduction of FOXM1 target genes. Furthermore, by overexpressing FOXM1 in PDAC cells we significantly reduced domatinostat-inducing oxidative mitochondrial and cellular stress and abolished GT sensitization, both in adherent and spheroid cells, confirming FOXM1 crucial role in the mechanisms described. Finally, we found a correlation of FOXM1 expression with poor progression free survival in PDAC chemotherapy-treated patients.

**Conclusions:**

Overall, we suggest a novel therapeutic strategy based on domatinostat to improve efficacy and to overcome resistance of commonly used chemotherapeutics in PDAC that warrant further clinical evaluation.

**Supplementary Information:**

The online version contains supplementary material available at 10.1186/s13046-022-02295-4.

## Introduction

Pancreatic ductal adenocarcinoma (PDAC) is still one of the most lethal cancers with reported 5-year relative survival rates ranging below 10%, representing the second largest cancer-related cause of the death and with incident rates on the rise [1]. Notably, more than 80% of patients with a diagnosis of PDAC present an advanced disease at diagnosis. Gemcitabine/nab-paclitaxel, represents a standard of care for unresectable or metastatic PDAC, however, the reported overall survival with this regimen, or with the alternative first line option FOLFIRINOX, remains less than 1 year [1].

Indeed, PDAC resistance to standard chemotherapeutics is a clinical challenge despite considerable efforts to improve clinical outcome. The lack of either effective targeted agents or immunotherapy approaches as well as a paucity of validated predictive biomarkers to guide therapeutic decision, make prompt the urgent need for new treatment options for this disease [1].

Cancer stem cells (CSC) are a small subset of cells characterized by self-renewal capability, distinctive metabolism and resistance to anticancer agents [2]. In several tumors, including PDAC, CSC have been identified as drivers of tumor growth and progression as well as the primary cause of resistance to conventional chemotherapeutics [3–7].

PDAC metabolic reprogramming to adapt and grow in a hypoxic environment due to the typical thick stroma is characterized, among others, by increasing oxidative metabolism resulting in increased generation of ROS by mitochondria [8]. Notably, within the tumor cells, the unique plasticity of CSC makes them particularly suitable to metabolic/oxidative stress adaptation [9].

Interestingly, the addiction of PDAC and of CSCs subpopulation to such metabolic pathways and to mitochondrial function might represent also an Achilles’ heel that can be therapeutically exploited [10].

Recently, the transcriptional factor FOXM1 has been found at elevated levels in patients with a bad prognosis in a multitude of malignancies, including pancreatic cancer [11]. There is already substantial evidence that FOXM1 plays key roles in a multiple range of biological processes, including cell proliferation, invasion, DNA damage repair, and stem cell renewal [12]. Moreover, FOXM1 as novel component of Wnt signaling pathway and as essential in the regulation of oxidative stress, contributed to malignant transformation and tumor cell survival [13].

Accumulating evidence suggests that epigenetic deregulation is a hallmark of cancer and has a major contribution to disease development, progression as well as resistance to antitumor treatment in several solid tumors, including pancreatic cancer. Histone deacetylase inhibitors (HDACi) are one of the most prominent classes of epigenetic drugs, we have been investigating as anticancer agents, both preclinically and clinically, for a long time [14]. Interestingly, it was reported that the antitumor effect of HDACi correlates with specific tumor epigenetic alterations, frequently associated with pancreatic cancer (i.e KDMA6 loss) [15]. Furthermore, HDACi have been demonstrated to target CSC subpopulation and to overcome drug resistance in several preclinical cancer models [14].

Domatinostat (4SC-202), is an orally administered small molecule class I selective HDACi and several trials are currently investigating this agent in combination with immunotherapies (ClinicalTrials.gov Identifier: NCT04874831, NCT04393753, NCT04871594, NCT04133948 and NCT03812796).

Domatinostat has been previously tested in vitro on two PDAC cell models demonstrating, as shown by other HDACi, the ability to counteract TGFβ-induced epithelial to mesenchymal transition, a described mechanism of chemoresistance, as well as to target CSC subpopulation [16, 17].

Here we demonstrated for the first time, both in vitro and in vivo in preclinical pancreatic cancer models, a significant synergistic antitumor effect of domatinostat in combination with the first line standard gemcitabine/nab-paclitaxel doublet chemotherapy treatment in PDAC patients. Moreover, we presented several evidence demonstrating that this effect is mediated by the down-modulation of FOXM1, leading to disruption of redox homeostasis and DNA repair, particularly in the CSC compartment, thus sensitizing PDAC cells to chemotherapy.

## Materials and methods

### Cell lines

The Human pancreatic cancer cell lines PANC1, ASPC1 and the hTERT-immortalized foreskin fibroblast BJhTERT were purchased from the American Type Culture Collection (ATCC, Rockville, MD, USA). PANC28 cell line was obtained from the laboratory of Dr. Marsha L. Fraizer and Dr. Douglas B. Evans. In adherent condition all cell lines were maintained as monolayer cultures and cultured in Dulbecco’s modified Eagle’s medium (DMEM) containing 4.5 g/L glucose, glutamine, and non-essential amino acids and supplemented with 10% heat-inactivated fetal bovine serum and penicillin (100 IU/mL)–streptomycin (100 μg/mL). In spheroid-forming condition all cell lines were plated (4000 cells/ml) in low attachment plates and cultured 48 h in sphere medium (DMEM / F12 supplemented with BSA 0.1%, glucose 0.5%, heparin 4 μg/ml, L-glutamine 2.5 mM, PS 1X, FGF 20 ng/ml, EGF 20 ng/ml, B27 1X, insulin 20 μg/ml) to perform the assays shown. Cultures were maintained in a humidified atmosphere of 95% air and 5% CO2 at 37 °C. All cell lines were regularly inspected for mycoplasma. The cells have been authenticated with short tandem repeat profile generated by LGC Standards.

### Reagents

All media, sera, antibiotics, and glutamine for cell culture were from Lonza (Basel, Switzerland). Primary antibodies for western blotting were used according to the manufacturer’s protocol: β-Actin (#8227), poly-(ADPribose)-Polymerase (PARP)-Ab (#556494), phospho-Histone H2AX (γH2AX) (#05636), FOXM1 (#5436S), β-catenin (#8480S), Oct-4 (#2750S), were purchased from Cell signaling Technology (Danvers, MA, USA). γ-Tubulin (#sc-7396) were purchased from Santa Cruz Biotechnology (Dallas, TX, USA). Secondary antibodies were purchased as follows: polyclonal goat anti-rabbit IgG (H + L)-HRP conjugate (#1706515) and polyclonal goat anti-mouse IgG (H + L)-HRP conjugate (#1706516) were purchased from Abcam (Cambridge, UK); polyclonal rabbit anti-goat IgG-HRP conjugate (#sc-2768) were purchased from Santa Cruz Biotechnology (Dallas, TX, USA). Goat polyclonal Secondary Antibody to Mouse IgG - H&L - Alexa Fluor® 594 (#ab150120). Stem cell viability was evaluated by 3D Cell Viability Assay (Promega, Madison, WI, USA) according to the manufacturer’s protocol.

### Drugs

Domatinostat (4SC-202) was obtained from 4SC AG (Planegg-Martinsried, Germany) and dissolved in sterile DMSO for in vitro experiments and in methylcellulose for in vivo experiments; gemcitabine (Accord, Devon, UK) and nab-paclitaxel (Celgene, Milan, Italy) were provided by our pharmacy. Taxol (#PHL89806), 5′-deoxy-5-fluoro-uridine, (5’DFUR) (#F8791), SN-38 (#sc-203,697), oxaliplatin (#O9512) were purchased from Sigma- Aldrich (St. Louis,MO, USA). Bortezomib was obtained from cell signal technology (Danvers, Massachusetts, USA). Stock solutions were diluted to appropriate concentrations in culture medium before addition to the cells.

### Cell proliferation assay and drugs combination studies

Cell proliferation was measured in 96-well plates in cells untreated and treated with described drugs as single agent or in combination. Cell proliferation was measured using a spectrophotometric dye incorporation assay (Sulforhodamine B) [18].

Drugs combination studies were based on concentration-effect curves generated as a plot of the fraction of unaffected (surviving) cells versus drug concentration after 96 h of treatment. Synergism, additivity, and antagonism were quantified after an evaluation of the CI, which was calculated by the Chou-Talalay equation with CalcuSyn software (Biosoft,Cambridge, UK), as described elsewhere [19]. A CI < 0.9, CI = 0.9–1.2, and CI > 1.2 indicated a synergistic, additive or antagonistic effect, respectively. The DRI determines the magnitude of dose reduction allowed for each drug when given in combination, compared with the concentration of a single agent that is needed to achieve the same effect.

### Clonogenic assay

Single cell suspensions were plated, as previously described [18] and treated or untreated with ≅ IC10 at 96 h concentrations of domatinostat (0.1 μM), gemcitabine (PANC1, 1.5 nM; PANC28, 5 nM; ASPC1, 1.5 nM) and Taxol (PANC1, 0.75 nM; PANC28, 0.15 nM; ASPC1, 0.325 nM). After 10 days, colonies were visualized and count as described previously [18].

### Limiting-dilution assay

Spheroid cultures, treated as indicated, were dissociated and live cells were sorted with a BD FACS Aria with a limiting dilution approach at 1, 2, 4, 8, 16, 32, 64 cells per well in ultra-low attached 96-well plates (Corning, NY, USA) in sphere medium. Stem cell frequency was evaluated after three weeks with the Extreme Limiting Dilution Analysis as described by Hu et al. [20].

### Cell cycle analysis

Cell cycle analysis was performed at the indicated times in all cell lines treated with domatinostat and GT, alone or in combination, as previously reported [21].

### Western blotting

Western blots were performed according to standard procedures [22]. Images were acquired using the Image Quant LAS 500 and the intensity was measured by Image Quant TL image software (GE Healthcare, Illinois, USA).

### RNA isolation, RT-PCR assays and real-time PCR

RNA was isolated by Trizol reagent (Invitrogen, CA, USA) as previously described [22]. Real-Time PCR by ABI Prism 7900 HT Sequence Detection System (Applied Biosystems, CA, USA) was performed using specific Taqman probes. All genes relative mRNA expression levels were calculated using the 2 ^-∆∆CT^ method and were normalized to that of β-actin as endogenous control gene β-actin.

### Immunofluorescence assay

6000 cell/well, plated on 96-wells, were treated with drugs as indicated in figure legends. Then cells were fixed in 4% paraformaldehyde (20 min at RT), blocked by 0.2% PBS/BSA solution (5 min at RT) and incubated with primary anti-FOXM1 antibody for 1 h at 37 °C. After washes, cells were incubated with anti-rabbit Alexa Fluor 595 (Thermo Fisher Scientific, Waltham, USA) overnight at 4 °C. Then the cells were washed and incubated 15′ with 4′,6-diamidin-2-fenilindolo (DAPI) (Thermo Fisher Scientific, Waltham, USA). Representative images were taken at 40X magnification by Opera Phenix microscope (PerkinHelmer,Waltham, MA USA) and the positive cells are counted by Harmony software (PerkinHelmer,Waltham, MA, USA).

### ROS production assays

ROS production was evaluated by culturing PANC1 and ASPC1 spheroids in Hydroethidine (Thermo Fisher Scientific, Waltham, MA, USA), according to the manufacturer’s protocol. Stained samples were evaluated by flow cytometry (FACS Canto, BD, Franklin Lakes, NJ, USA) and results analyzed with BD FlowJo software. The production of superoxide by mitochondria was evaluated by the fluorescent MitoSOX™ Red reagent (Thermo Fisher Scientific, Waltham, MA; USA), according to the manufacturer’s protocol and by microscopy. Briefly, PANC1 and ASPC1 spheroids were dissociated and plated on cover slips. Subsequently, the cells were treated as reported in figure legend with domatinostat (0.5 μM). Then, the media was removed and pre-warmed (37 °C) staining solution containing MitoSox probe was added for 15 min at 37 °C. The staining was photographed by Opera Phenix microscope (PerkinHelmer,Waltham, MA USA) and the positive cells are counted by Harmony software (PerkinHelmer,Waltham, MA, USA).

### Chromatin immunoprecipitation (ChIP)

PANC-1 cells (12X10^6^) were crosslinked at room temperature for 10 min by adding formaldehyde to a final concentration of 1%. The action of formaldehyde was then neutralized by adding freshly dissolved Glycine to a final concentration of 125 mM. Cells were then centrifuged at 1200 rpm for 5 min and the pellet was washed twice in 1X cold PBS. Subsequently, cells were suspended in lysis buffer B (20 mM HEPES pH 7.7, 10 mM EDTA, o,5 mM EGTA, 0,25% triton X100) and C (50 mM HEPES pH 7.6, 150 mM NaCl, 1 mM EDTA, 0,5 mM EGTA) and alternately placed on a wheel at 4°C for 10 min. Another centrifugation was performed to collect the nuclei in buffer D (20 mM HEPES ph 7.6, 1 mM EDTA, 0,5 mM EGTA, 0,05% SDS and protease inhibitors). The nuclei were sonicated at maximum intensity by using Bioruptor Next Gen (Diagenode, Ougrée, Belgium). After sonication, samples were centrifuged at 13000 rpm for 10 min at 4°C and the supernatants were collected for ChIP assay analysis. The supernatants were transferred to a clean tube where the antibody was also added (about 3 μg per reaction). Immunoprecipitation was performed overnight at 4°C on a wheel and by adding to supernatant Protein A/G PLUS (Santa Cruz Biotechnology, Dallas, TX, USA, sc-2003), antibody, incubation buffer 1X (10 mM Tris pH 8, 150 mM NaCl, 1 mM EDTA, o,5 mM EGTA, o,15% SDS, 1% Triton X-100, protease inhibitors and 0,1% BSA). In parallel 10% of each sample was taken as an input indicator for further PCR analysis. The following day, samples were centrifugated at 1200 rpm for 5 min at 4°C and the supernatant was removed with several washes of 10 min at 4°C, using 500 μL of each wash buffer. After the final wash, 400 μL of elution buffer were added. Elution was carried out for 30 min at room temperature on a wheel. Subsequently, 125 mM NaCl was added to 400 μL of the sample. De-crosslinking continued overnight at 65°C. The day after, proteins were degraded by treatment with proteinase K, performed by incubating with 0.5 M EDTA, 1 M Tris pH 6.5 and proteinase K for 1 h at 45°C. DNA was then recovered with MinElute Reaction Cleanup Kit (Qiagen, Hilden, Germany). Real-time PCR analysis was then performed on these samples. The antibody used for this assay was: Anti-FOXM1 (Millipore, Burlington, MA, Stati Uniti). The following gene promoters were used: SOX2 FW: 5′-AGGGAGAGAAGTTTGAGCCC-3′; SOX2 REV:5′-GCGAGGAAAATCAGGCGAAG-3′; Rad51 FW:5′-GTAAAACTTGGCCCCTACACTG-3′; Rad51 REV:5′-ATAAGGTGCATCTCTCTCCCC-3′; OCT4 FW: 5’TGGAGGTGTGGGAGTGATTC-3′;OCT4 REV: 5-GACTACAGGCTTGGACCACT-3′; BIRC5 FW: 5′-TTTGCGAAGGGAAAGGAGGA-3′; BIRC5 REV: 5′-AATGAACAGGGGAGGGATGG-3′;CAT FW: 5′-TGGTCTACTTTGCAAGCTTGG-3′; CAT REV:5- AAGGTAATTGCAAGTGATTGGTT-3′; XRCC1 FW: 5′-GCGGGCGTAGTAAAAGACAG-3′; XRCC1 REV: 5′-TGAGGCCAAAAGAGAAGGGT-3′.

### Apoptosis analysis

PANC1 and ASPC1 spheroid cultures were treated as reported in figure legends with domatinostat and/or NAC (Sigma-Aldrich, St. Louis, MO, USA) or Mitoquinone mesylate (MitoQ) (Selleck Chemicals LLC, Houston, TX, USA). Cells were then dissociated and stained with CD133-APC antibody (1:100, MiltenyiBiotec, Paris, France) in PBS for 20 min at 4°C, washed with PBS and re-suspended in PBS-Annexin V-FITC from BD (BD, Franklin Lakes, NJ, USA) for 15 min at 4°C for evaluation of apoptotic cells.

### Network analysis

A network analysis was generated by (ingenuity pathway analysis) ipa software (GeneGo Inc., St. Joseph, MI, USA). IPA includes a manually annotated database of protein interactions and metabolic reactions obtained from the scientific literature. The networks were graphically visualized as hubs (proteins) and edges (the relationship between proteins).

### Plasmide transfection

The pCW57.1-FOXM1c plasmids were purchased from Addgene (Watertown, MA, USA). Adherent PANC1 cells were transfected using Lipofectamine 2000 Reagents (Thermo Fisher Scientific, Waltham, MA, USA), according to the manufacturer’s recommendation. After 4 h from transfection, cultures were used for western blot, real-time PCR, immunofluorescent experiments, cell survival assay as described above.

### Genomics analysis

Genomics analysis were performed taking advantage of web servers for analyzing RNA sequencing expression data from the TCGA http://gepia.cancer-pku.cn/ and http://r2.amc.nl.

### In vivo xenograft studies

All studies have been performed in accordance with the institutional guidelines and approval by local authorities (377/2019), in line with “Directive 2010/63/EU on the protection of Animals used for scientific purposes” and made effective in Italy by the Legislative Decree DLGS 26/2014.

PANC28 and PANC1 cells were respectively suspended in 5*10^6^ cells in 200 μl of PBS and Matrigel (BD Pharmingen, Milan, Italy) (1:1) and 5*10^6^ cells in 200 μl of PBS. The cells were subcutaneously injected in the flanks of 6-week-old female nude mice (Envigo Laboratories, Indianapolis, IN, USA). The mice were acclimatized in the Animal Care Facility of CROM–Centro Ricerche Oncologiche di Mercogliano. Tumor volume [1/2(length × width^2^)] was assessed using digital caliper. When the tumors became palpable, the mice were randomized into four experimental groups (*n* = 7). Mice were treated as followed: (a) vehicles; (b) gemcitabine (weekly 25 mg/Kg, i.p.) and nab-paclitaxel (weekly 20 mg/Kg, i.p.) re-suspended in salt solution 100 μl per dose; (c) domatinostat (20 mg/Kg 5 days/week, per os) re-suspended in Methocel 2% solution 250 μl per dose; (d) triple combination gemcitabine/nab-paclitaxel plus domatinostsat. Drug treatments were administered for 3 weeks. All mice received drugs vehicles. TGD and the percent change in the experimental groups was compared with that of the vehicle control groups as described before [23].

### Immunohistochemistry on xenograft tumor samples

Both expression and localization of β-catenin were evaluated by IHC on formalin fixed paraffin embedded tumor samples derived from mice sacrificed at the end of PANC1 in vivo experiment. Briefly, the sections were incubated with primary antibody and then with biotin-conjugated secondary antibody, before incubation with specific streptavidin HRP-conjugated tertiary antibody (Thermo Fisher Scientific, Waltham, MA USA). Peroxidase reactivity was visualized using a 3,3′-diaminobenzidine (Abcam, Cambridge, UK). A single pathologist (R D.C.) performed a blinded analysis of the slides.

### Statistical analysis

All experiments were performed at least three times. Statistical significance was determined by the one-way ANOVA and Tukey Test and a *p* < 0.05 was considered to be statistically significant. All statistical evaluations were performed with Graph Pad Prism 7.

## Results

### Domatinostat sensitizes pancreatic cancer cells to chemotherapy

**Fig. 1:**
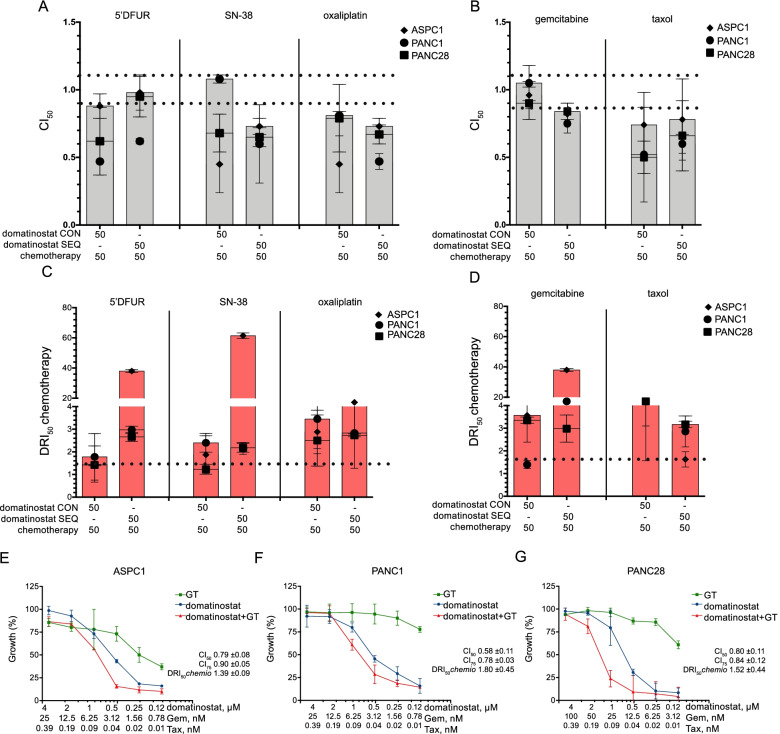
Domatinostat can sensitize pancreatic cancer cells to chemotherapeutics treatment. CI (combination index) values (mean ± SD from at least three separate experiments performed in quadruplicates) computed at 50% of cell kill (CI_50_) CalcuSyn software after 96 h. Two schedules of treatment were represented in PANC1, PANC28 and ASPC1 cell lines. Equitoxic doses of drugs with domatinostat administered at same time with chemotherapy (in figure domatinostat CON) and equitoxic doses of drugs with domatinostat administered 24 h before the same chemotherapy (in figure domatinostat SEQ). In **A**. 5’DFUR, SN-38 and oxaliplatin plus domatinostat combinations were reported, in **B**. gemcitabine and taxol plus domatinostat combinations were reported. The combinations were considered synergistic when CIs were below 0.9 and additive when CIs were below 1.1 **C**.**-D**. DRI (doses reduction index) values (mean ± SD from at least three separate experiments performed in quadruplicates) computed at 50% of cell kill (DRI_50_) CalcuSyn software after 96 h in PANC1, PANC28 and ASPC1 cells. In C. DRI_50_ for 5’DFUR, SN-38 and oxaliplatin plus domatinostat combinations. In **D**. DRI_50_ for gemcitabine and taxol plus domatinostat combinations. **E**.**-F**. ASPC1, PANC1 and PANC28 cells were treated for 96 h with increasing concentration of domatinostat alone and in combination with increasing concentration of Gemcitabine/Taxol (GT) (doses are reported in figure). Cell growth expressed as percentage of control was assessed by sulforhodamine B colorimetric assay (see Materials and Methods)

In order to explore the potential of domatinostat as an effective therapeutic approach to sensitize PDAC to chemotherapy, we performed an in vitro screening of drug combinations using chemotherapeutic agents currently employed for PDAC treatment, such as fluoropyrimidines (evaluating 5’DFUR, an intermediary prodrug of 5-fluorouracil), irinotecan (evaluating the active metabolite SN-38), oxaliplatin, gemcitabine and taxol. We tested three different pancreatic cancer cell lines (PANC1, PANC28 and ASPC1), showing a high similarity in domatinostat sensitivity, compared to striking differences in sensitivity to chemotherapeutics, tested in monotherapy (Suppl. Table S1 and Suppl. Fig. S1). We explored different cytotoxic ratios of domatinostat in combination with chemotherapeutic agents, either at equipotent doses (50:50 ratio) or using lower doses of chemotherapeutics (75:25 ratio), either simultaneously or sequentially (with a 24 h delay between the two agents). The combination index (CI) values, calculated at 50% (CI_50_) of cell lethality demonstrated mostly synergistic (CI < 0.9) or additive (CI < 1.1) effects with all the anticancer agents tested, in all three cell lines (Fig. 1A-B and Suppl. Tables S2–5). Concomitant or sequential treatments were equally effective, although, CI values differ depending on the chemotherapeutic employed. Interestingly the synergistic interaction was also observed using lower doses of chemotherapeutics (75:25 ratio) (Suppl. Tables S3 and S5). Indeed, the evaluation of the dose reduction index (DRI) values, which represent the order of magnitude (fold) of dose reduction, obtained for the IC_50_ (DRI_50_) in combination treatment compared with single-drug treatment, confirmed, for all the chemotherapeutics evaluated, a significant potentiation of the antitumor effect when combined with domatinostat, in all three cell lines, with both simultaneous and sequential schedules (Fig. 1C-D, Suppl. Tables S2–5).

Next we explored combination treatment of domatinostat plus chemotherapy doublets, observing again significant synergistic anti-proliferative effects, either with gemcitabine/taxol (GT) (Fig. 1E-G and Suppl. Tables S2–3) or with fluoropyrimidine/irinotecan (5’DFUR/SN38) combinations (Suppl. Tables S4–5). Notably, in the hTERT-immortalized foreskin fibroblast BJhTERT cells, a non-tumorigenic cell line, we observed antagonist effects in all drug combinations tested (Suppl. Tables S2–5), suggesting a selective synergistic effect of domatinostat plus chemotherapy in tumor cells.

Since GT is the most common first-line option for the treatment of metastatic PDAC patients we further investigated the mechanism underlying the observed synergism by using domatinostat plus this chemotherapy doublet. Notably, for all the following experiments, if not differently mentioned, we tested domatinostat at 0.5 μM, a low dose if compared with reported preclinical studies with this agent [17, 24–27].

**Fig. 2:**
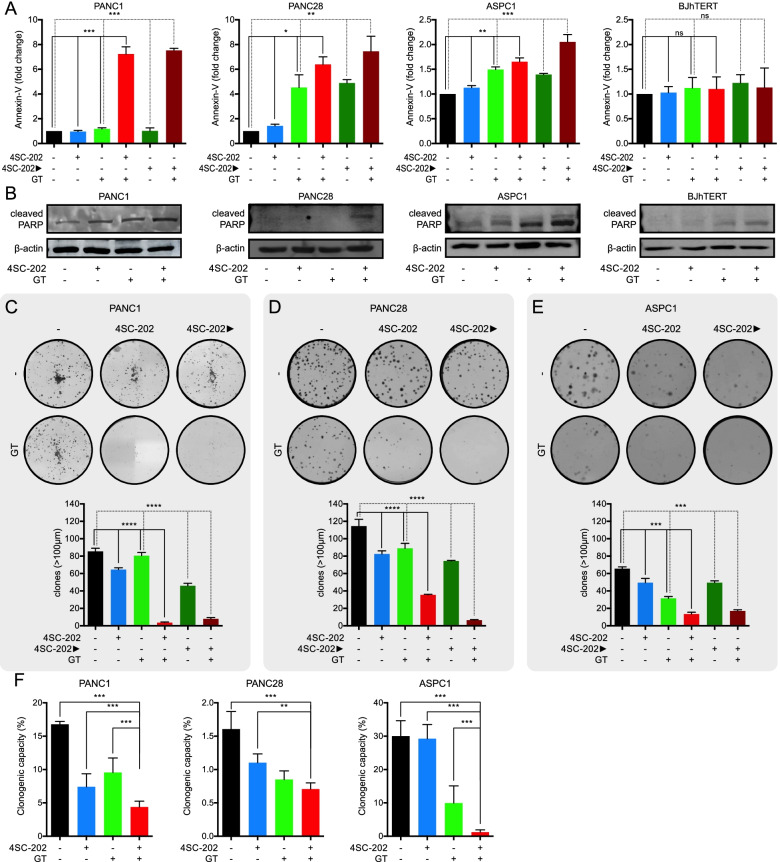
Combination of domatinostat and gemcitabine/taxol induces apoptosis and reduces clonogenic capacity in PDAC cells. **A.** Flow cytometry analysis of Annexin-V shows the outcome of the induced chemosensitivity in PANC1, PANC28, ASPC1 and BjhTERT (human-derived fibroblast) cells when domatinostat (0.5 μM) is combined with gemcitabine/taxol (GT) (IC_50_ at 96 h) for 24 h. **B.** Apoptosis, evaluated by PARP cleavage induction. In PANC1, PANC28, ASPC1 cells PARP cleavage is induced when the cells are treated with domatinostat (0.5 μM) plus GT (IC_50_ at 96 h) for 48 h. **C-D-E.** Clonogenic assay shows the long-term effects of combination treatment domatinostat (0.1 μM) plus GT (IC_10_ at 96 h) in PDAC cell lines collected 10–15 days after treatment. A one well picture in a representative experiment is shown for each treatment; bar graphs show the numbers of colonies for each condition (mean ± SD of 2 or more separate experiments each one with technical triplicate). **F.** Limiting dilution assay performed on PDAC cells, untreated or treated for 24 h with domatinostat (0.1 μM) plus GT (IC_10_ at 96 h) and plated in ultra-low 96-well without additional treatment for three weeks. Clonal frequency was evaluated with the Extreme Limiting Dilution Analysis ‘limdil’ function as described in Material and Methods section. Data are mean ± SD; *n* = 3 independent experiments, analyzed using two way ANOVA test. **P ≤ 0.01, ***P ≤ 0.001

We first confirmed the synergistic antitumor interaction by demonstrating in all three PDAC cell lines a clear statistically significant potentiation of apoptosis in combination treatment as compared to domatinostat or GT alone (tested at IC_50_^96h^), as shown by Annexin-V staining (Fig. 2A and Suppl. Fig. S2) and PARP-cleavage (Fig. 2B). These effects were not observed in BJhTERT cells. Accordingly, the synergistic pro-apoptotic effect was paralleled by a cell cycle perturbation effect characterized by a S-phase block induced by triple combination after 48 h of treatment in PDAC cells lines but not in normal BJhTERT cells (Suppl. Fig. S3).

Taking advantage of colony formation assay, we then evaluated domatinostat plus GT (IC_10_^96h^), either simultaneously or sequentially (with a 24 h delay between the two agents) on the three PDAC cell lines. As shown in Fig. 2C-E, we demonstrated a dramatic reduction of colony formation by triple domatinostat/GT combination in both treatment schedules, as compared to control, domatinostat or GT alone.

Finally, to determine the effect of domatinostat plus GT (IC_10_^96h^), on CSC compartment, we performed a limiting dilution assay. Notably, we demonstrated a statistically significant reduction of clonogenicity of PANC1, PANC28 and ASPC1 cells upon incubation for 24 h with triple combination vs domatinostat or GT alone (Fig. 2F).

Overall these data demonstrated the ability of domatinostat to sensitize pancreatic cancer cells to different chemotherapeutic agents employed in clinical practice to treat PDAC patients.

### Domatinostat potentiates chemotherapy by targeting CSC compartment through reactive oxygen species accumulation and stress-induced apoptosis

In order to better define the potential impact on CSC compartment we next explored the effect of domatinostat alone, or in combination with GT, on PDAC cell-derived self-assembled spheroids, a model characterized by a clear CSC-enrichment (Suppl. Fig. S4).

**Fig. 3:**
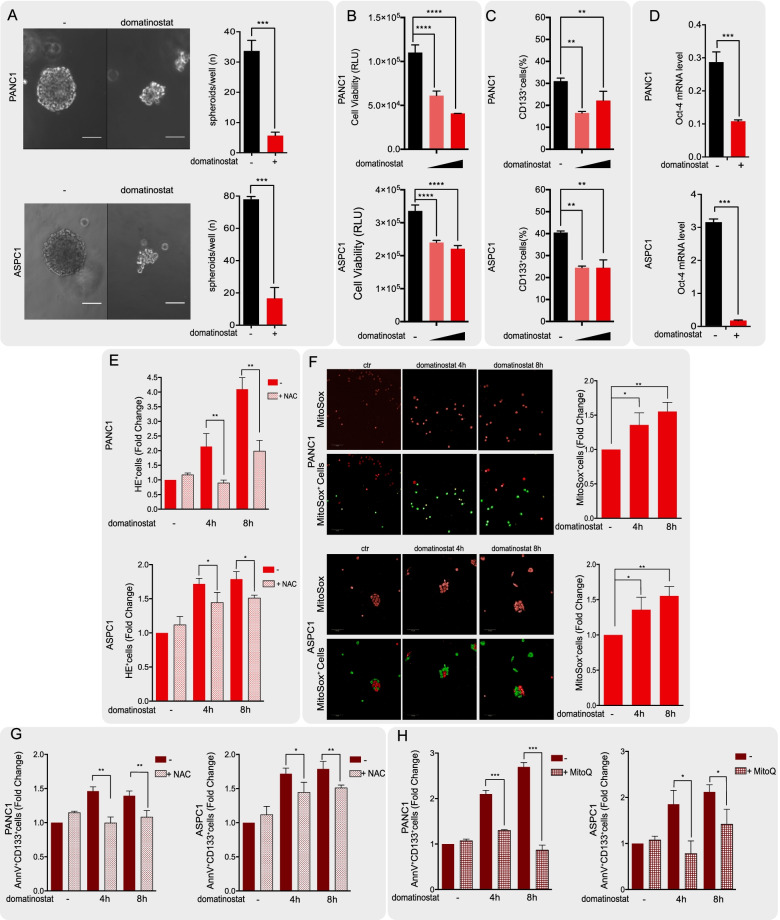
Domatinostat affects PDAC stem cells by modulating oxidative stress. **A.** The effect of domatinostat (0,5 μM) on PANC1 and ASPC1 spheroid cultures. Cells (1000/mL) seeded in a matrigel drop and sphere medium, were treated with and without domatinostat and collected 7 days after treatment. Images of one spheroid for each condition in a representative experiment is shown (white scale bar: 50 μm, magnification 20X). On the right, bar graphs show the numbers of spheroids for well (mean ± SD of 2 or more separate experiments each one with technical triplicate). **B.** PANC1 and ASPC1 spheroids viability treated with and without domatinostat (0.5 μM and 1 μM) was assessed by cell titer luminescence assay (see Materials and Methods) (mean ± SD of 2 or more separate experiments each one with technical triplicate). **C.** Flow cytometry assay shows CD133 protein expression decrease after domatinostat (0.5 and 1 μM) treatment for 16 h in PANC1 and ASPC1 cells. **D.** qRT-PCR analysis shows Oct-4 levels drop when PANC1, and ASPC1 spheroids are treated with domatinostat (0.5 μM) for 16 h. **E.** Cellular ROS production is visualized by Hydroethidine (HE) staining. PANC1 and ASPC1 spheroids were treated with domatinostat (0.5 μM) alone and in combination with N-acetylcysteine (NAC, 5 mM), as ROS scavenger, at the indicated timing. Cells were stained for HE as described in Material and Methods section and visualized by flow cytometry. **F.** Mitochondrial ROS amount is analyzed by mitosox staining. PANC1 and ASPC1 spheroids treated with or without domatinostat (0.5 μM) alone at the indicated timing were fixed, stained for mitosox (red) and measured by Opera Phenix confocal microscopy. The mitosox positive cells are counted by Harmony software as described in Material and Methods section. Representative images (20X magnification) show stained cells (red) and mitosox counted positive cells (green). **G.-H.** The observed increase in ROS amount is related to an increase of apoptotic cancer stem cells upon domatinostat treatment. PANC1 and ASPC1 spheroids, treated as previously, were stained for AnnexinV-FITC and CD133-APC as described in Material and Methods section and visualized by flow cytometry. In **G.** PANC1 and ASPC1 spheroids were treated with domatinostat (0.5 μM) alone and in combination with NAC, 5 mM, as ROS scavenger, at the indicated timing. In **H.** PANC1 and ASPC1 spheroids were treated with domatinostat (0.5 μM) alone and in combination with Mitoquinone mesylate (MitoQ) 100 nM, as mitochondrial ROS scavenger, at the indicated timing. (Statistically significant results by ANOVA test are reported *** indicates *P* < 0.0001, ** indicates *P* < 0.005 and * indicates *P* < 0.05)

First, we observed that domatinostat alone, at low doses (0.5 μM and 1 μM), strongly reduced size, amount and viability of PANC1, PANC28 and ASPC1 spheroids (Fig. 3A-B, Suppl. Fig. S5A-B, Suppl. Fig. S6). Notably, we also demonstrated a clear reduction of CSC marker CD133 surface expression (Fig. 3C and Suppl. Fig. S5C) as well as of CD133 mRNA levels (Suppl. Fig. S5D) within 16 h of domatinostat treatment in all PDAC spheroids. Similarly, a reduction of CSC marker Oct-4 mRNA level was also demonstrated in all three spheroid models after 24 h of treatment with domatinostat (Fig. 3D and Suppl. Fig. S5E).

Furthermore, since it has been reported that oxidative stress modulation has a critical role in CSC by promoting proliferation, adaptation and resistance to chemotherapy [9], we also investigated the effects of domatinostat on CSCs redox homeostasis. In both PANC1 and ASPC1 spheroids we demonstrated a time-dependent increase in total ROS cellular amount induced by domatinostat within 4 h of treatment, reverted by concomitant treatment with the ROS scavenger N-acetylcysteine (NAC) (Fig. 3E). This effect was confirmed by concomitat ROS accumulation in mitochondrial compartment, the main source of ROS in living cells, as demonstrated by mitosox IF staining (Fig. 3F). Domatinostat-mediated ROS accumulation was paralleled within the same time frame by a pro-apoptotic effect in CSC subpopulation, as shown by Annexin V staining of CD133 positive cells. Notably, this effect was partially reverted by NAC, indicating that the induction of apoptosis was at least in part due to ROS accumulation (Fig. 3G). Intriguingly, apoptotic effect induced by domatinostat was almost completely reverted by concomitant treatment with the mitochondria ROS scavenger mitoquinone mesylate (MitoQ) (Fig. 3H). Overall, by comparing the efficacy of MitoQ vs NAC in reverting apoptosis, we can speculate that CSC are more sensitive to mitochondria ROS levels alteration comparing to cytoplasmic ROS levels.

**Fig. 4:**
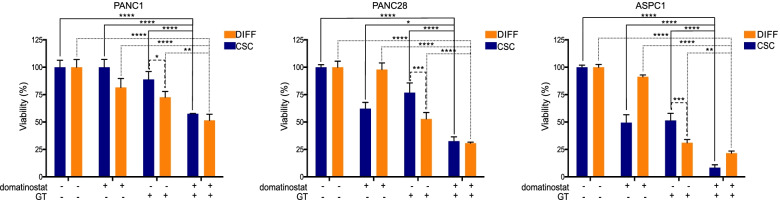
Domatinostat sensitizes both PDAC stem cells and differentiated cells to chemotherapy. PANC1, PANC28 and ASPC1 cells were growing in differentiation condition (DIFF in blue) and as spheroids (CSCs in orange), then are treated for 72 h with domatinostat (1 μM) alone and in combination with gemcitabine/taxol (respectively, 12.5 nM and 1 nM). Cell growth expressed as percentage of control was assessed by Cell titer assay (see Materials and Methods). Statistically significant results by ANOVA test are reported (*** indicates *P* < 0.0001, ** indicates *P* < 0.005 and * indicates *P* < 0.05)

Finally, we tested domatinostat in combination with GT in PANC1, PANC28 and ASPC1 spheroid models as compared to the differentiated counterpart cells grown in adherent conditions. Notably, spheroid models are significantly more resistant to GT treatment (G:12.5 nM; T: 1 nM; 72 h), however in combination with domatinostat (1 μM) we observed a similar significant decrease in viability in both spheroids and differentiated cells (Fig. 4). Interestingly, in two out of three cell lines (PANC28 and ASPC1) domatinostat alone was even more effective on spheroids compared to adherent growing cells.

Overall, these data demonstrated that domatinostat has an efficient antitumor effect on PDAC spheroids and thus on CSC subpopulation, related, at least in part, to the alteration of CSC mitochondrial and cellular oxidative homeostasis. Moreover, we might argue that the selective effect of domatinostat on CSC could be responsible for the clear potentiation of GT antitumor effect observed in combination treatment.

### Domatinostat induced antitumor effect and sensitization to chemotherapy by altering redox homeostasis of CSC via down-modulation of the transcription factor FOXM1

**Fig. 5:**
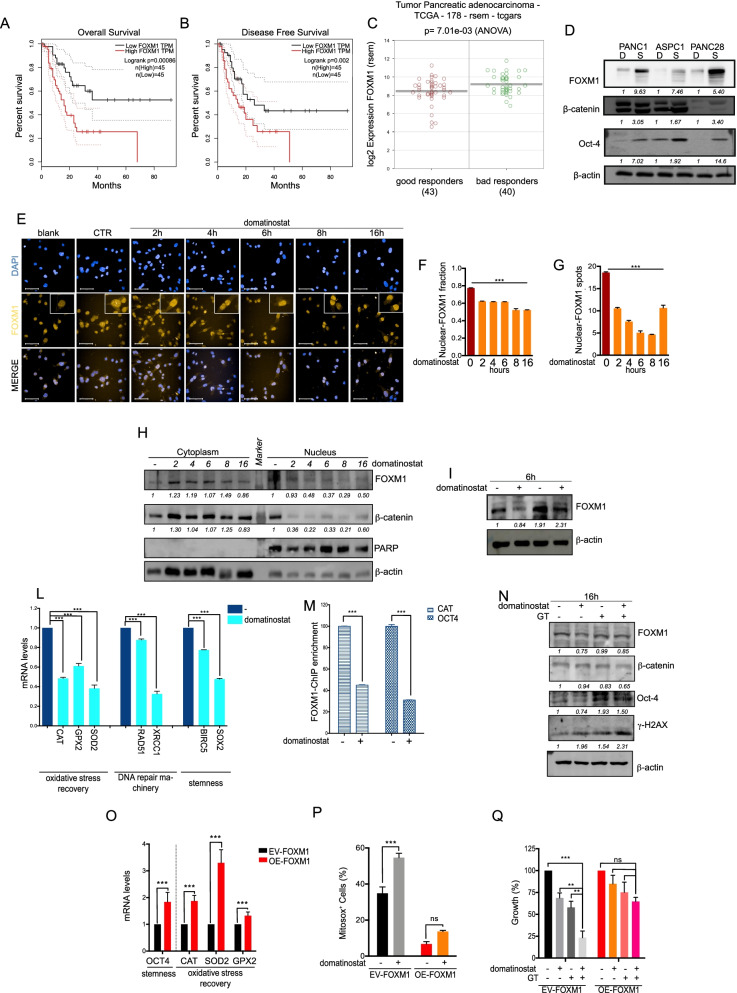
Domatinostat potentiates chemotherapy effect by modulating expression and localization of FOXM1 in CSCs. **A**. FOXM1 expression in patients with poor (dead; *n* = 93) and good prognosis (alive; *n* = 85) in the TCGA PAAD cohort (Wilcox-test W = 2647, *p*-value< 0.00058). **B**. FOXM1 expression in sensitive and resistant to the primary therapy in the TCGA PAAD cohort, evaluated as PFS (Wilcox-test W = 2269, *p*-value< 0.00012). **C.** FOXM1 expression in good (complete-remission_response patients, *n* = 43) vs bad (progressive-disease patients, *n* = 40) responders to chemotherapy, picked in “treatment_outcome_first_course” subset patients (One Way Analysis of variance; *p*-value< 0.000701). **D.** Basal protein levels of FOXM1 and stem-cell markers (β-Catenin; Oct-4) in PANC1, PANC28 and ASPC1 spheroids (S) versus differentiated cells (D). β-actin serves as loading protein control. **E.** Nuclear localization by IF upon domatinostat (0.5 μM) treatment in PANC1 spheroid cells at indicated timing. Bar 50 μM. Magnification 40X. DAPI is for nuclear staining. **F.** FOXM1-nuclear intensity quantified by Harmony software. **G.** FOXM1-nuclear spots were quantified by Harmony software. **H.** WB analysis of nuclear and cytoplasmic FOXM1 in PANC1 spheroids treated with domatinostat (0.5 μM) at the indicated timing. PARP and β-actin serve as nuclear and cytoplasmic loading control, respectively. **I.** FOXM1 protein expression in PANC1 spheroids, treated with domatinostat (0.5 μM) and domatinostat plus Bortezomib (20 mM) for 6 h. β-actin serves as loading control. **L.** CAT, GPX2, SOD2, RAD51, XRCC1, BIRC5 and SOX2 mRNA levels in PANC1 spheroids, treated with domatinostat (0.5 μM) for 16 h. **M.** ChIP-qPCR analysis showing the relative decrease of enrichment of FOXM1 binding to CAT and OCT4 promoters. Data obtained on immunoprecipitated fractions were normalized to input chromatin (IP/Input). The mean of at least two independent experiments with error bars indicating the SD. **N.** FOXM1, β-Catenin, Oct-4 and γH2AX protein expression in PANC1 spheroids treated for 16 h with domatinostat, GT (IC_50_^96h^) or their combination. β-actin serves as loading control. **O**. OCT4, CAT, SOD2 and GPX2 mRNA levels in PANC1 cells transfected with FOXM1 (OE-FOXM1) or empty vector (EV-FOXM1). **P.** Mitochondrial ROS amount in OE-FOXM1 and EV-FOXM1 PANC1, treated or untreated with domatinostat (0.5 μM) for 16 h, visualized by mitosox staining. **Q.** OE-FOXM1 and EV-FOXM1 PANC1 cells were treated for 96 h with domatinostat (1 μM) alone or in combination with GT (respectively, 100 nM and 1.56 nM). Cell growth expressed as percentage of control was assessed by SRB colorimetric assay. The values are the means ±S.D. from at least three independent experiments. Statistically significant results, by 2-way ANOVA test, are reported (***indicates *P* < 0.0001, **indicates *P* < 0.005 and *indicates *P* < 0.05)

To better understand the molecular mechanism by which domatinostat induces a ROS-mediated cell-death in CSC, we performed RNA-seq data-mining from published results of domatinostat effects on PANC1 cells [17]. In details, we analyzed the expression of oxidative-stress related genes whose high expression we have previously demonstrated to be statistically significant associated with poor prognosis in solid tumors, including pancreatic cancer [9]. Among these genes, FOXM1 emerged as the top strongly down-regulated gene by domatinostat treatment (Suppl. Table. S6–7). FOXM1, is a transcription factor with several functions, that has been reported to play a critical role in pancreatic cancer [9]. Indeed, by analyzing TCGA expression data in pancreatic cancer (PAAD dataset) we evidenced that FOXM1 high level is significantly related with bad overall survival (Fig. 5A), disease free survival (Fig. 5B) and with chemotherapy response (Fig. 5C) in PDAC patients.

We also found a higher FOXM1 protein expression in PANC1, PANC28 and ASPC1 spheroid models compared to differentiated cells, correlated with high β-Catenin and Oct-4 protein levels, thus suggesting and enrichment of FOXM1 expression in CSC subpopulation (Fig. 5D). Indeed FOXM1 governs the recruitment of β-catenin to the β-catenin-TCF4 transcription activation complex in the Wnt target gene promoter thus being involved in CSC phenotype [28]. We confirmed that domatinostat treatment leads to significant decrease of FOXM1 mRNA expression in all three PDAC cell lines (Suppl. Fig. S7A).

We next investigated FOXM1 localization upon domatinostat treatment from 2 up to 16 h, in PANC1 spheroids, demonstrating, by IF staining (Fig. 5E), a time-dependent decrease in nuclear FOXM1 localization with a peak between 6 and 8 h of treatment, reported also as decrease of nuclear-FOXM1 fraction and nuclear-FOXM1 spots (Fig. 5F and Fig. 5G). Furthermore WB analysis confirmed FOXM1-nuclear reduction, paralleled by protein cytoplasmic accumulation (Fig. 5H), thus suggesting that domatinostat affects FOXM1 activity also preventing its nuclear translocation. Notably, we observed a simultaneous similar β-catenin cytoplasmic localization following domatinostat treatment (Fig. 5H), confirming the tight functional connection between FOXM1 and β-catenin.

Moreover, since it has been previously demonstrated that FOXM1 protein levels is also regulated by ubiquitination and deubiquitination process and, thus, proteasome-dependent degradation [29], we evaluated domatinostat effect on FOXM1 protein expression in absence or presence of the proteasome inhibitor, bortezomib. As shown in Fig. 5I, concomitant treatment with bortezomib, completely reverted domatinostat-mediated inhibition of FOXM1 protein, suggesting that the inhibition of FOXM1 observed in PANC1 is partially due to domatinostat-increased FOXM1 protein degradation within 6 h from treatment (Fig. 5I).

Notably, domatinostat-mediated FOXM1 decrease was paralleled by the reduction of FOXM1 transcriptional target genes such as the oxidative stress-response antioxidants CAT, GPX2 and SOD2, the DNA damage-related RAD51 and XRCC1 genes and the stemness-related genes BIRC5 and SOX2 (Fig. 5L). Then, we performed a ChIP assay to investigate if domatinostat was able to reduce the FOXM1-binding to promoters of recovery stress (CAT), stemness (OCT4, BIRC5 and SOX2) and DNA damage (RAD51 and XRCC1) genes that we found as down-regulated at transcriptional level by domatinostat. In line with downregulation at transcriptional level, ChIP experiments using PANC1 spheroids followed by semiquantitative and quantitative PCR (Fig. 5M and Suppl. Fig. S7B) revealed the presence of FOXM1 on CAT, OCT4, BIRC5, SOX2, RAD51 and XRCC1 promoters in untreated conditions and its displacement after 16 h of treatment with domatinostat (1μM). Moreover, FOXM1 as well as β-catenin protein levels were reduced by domatinostat alone or in combination with GT (IC_50_^96h^) in PANC1 spheroids (Fig. 5N) and PANC28 and ASCP1 spheroids (Suppl. Fig. S8). This effect was paralleled by synergistic induction of DNA damage in triple combination as compared with domatinostat or GT alone, as demonstrated by increased expression of γ-H2AX (Fig. 5N and Suppl. Fig. S8).

To further confim the molecular mechanism behind the ability of domatinonstat to modulate stemness and oxidative stress homeostasis we performed an ingenuity pathway analysis (IPA) search on “BIRC5, NANOG, POUF1, CTNNB1 and SOX2”, as domatinostat modulated-stemness markers, and “GPX2, CAT, SOD2, RAD51 and XRCC1”, as domatinostat modulated-stress markers. IPA network revealed direct relationships between all the protein used as input, confirming a functional relationship between the targets of our treatment combination. Moreover, FOXM1 came out in the IPA upstream analysis as the most significant upstream regulator (Suppl. Fig. S9). Furthermore, an analysis on TCGA-PAAD data demonstrated a strong positive expression pattern correlation between FOXM1 and either DNA damage-related genes, such as EXO1, RAD51, XRCC2 and a stemness related gene, such as BIRC5 and the FOXM1-specific deubiquitinase, USP5 (Pearson’s R < 0.65) in pancreatic tumor tissues (Suppl. Table S6 and S7 and Suppl. Fig. S10), overall confirming and reinforcing our observations.

To confirm that the antitumor effect of domatinostat alone and in combination with GT is mechanistically connected with the modulation of FOXM1 and oxidative stress in CSC subpopulation, we then generated transiently FOXM1 over-expressing PANC1 cells (OE-FOXM1) (Suppl. Fig. S11). Notably, OE-FOXM1 cells showed higher mRNA levels of CSC marker Oct-4 and of oxidative stress-response FOXM1 transcriptional targets CAT, SOD2 and GPX2, compared to empty vector-transfected cells (EV-FOXM1) (Fig. 5O). Moreover, the cellular (Suppl. Fig. 12A) and mitochondrial (Fig. 5P) ROS accumulation induced by domatinostat was significantly reduced or completely abolished, respectively, in OE-FOXM1 compared to EV-FOXM1 cells. Notably, basal mitochondrial ROS levels in OE-FOXM1 cells were dramatically reduced, confirming a critical antioxidant role of FOXM1, particularly in the mitochondrial compartment.

Finally, coherently with the data presented above, OE-FOXM1 cells were less sensitive to either domatinostat or GT compared to EV-FOXM1 cells, and, more importantly, the synergistic antitumor effect of the triple combination was abolished in OE-FOXM1 cells (Fig. 5Q, Suppl. Fig. S12B).

Overall, FOXM1, a transcription factor correlated with PDAC patients’ bad prognosis, appears to play a critical role in the redox homeostasis of PDAC cells particularly in the CSC compartment. Indeed, FOXM1 down-modulation by domatinostat induced ROS accumulation targeting CSC subpopulation, thus leading to antitumor effect and sensitization to chemotherapy.

### In vivo synergistic antitumor effect of domatinostat plus gemcitabine/nab-paclitaxel in PANC1 and PANC28 xenograft mouse models

**Fig. 6:**
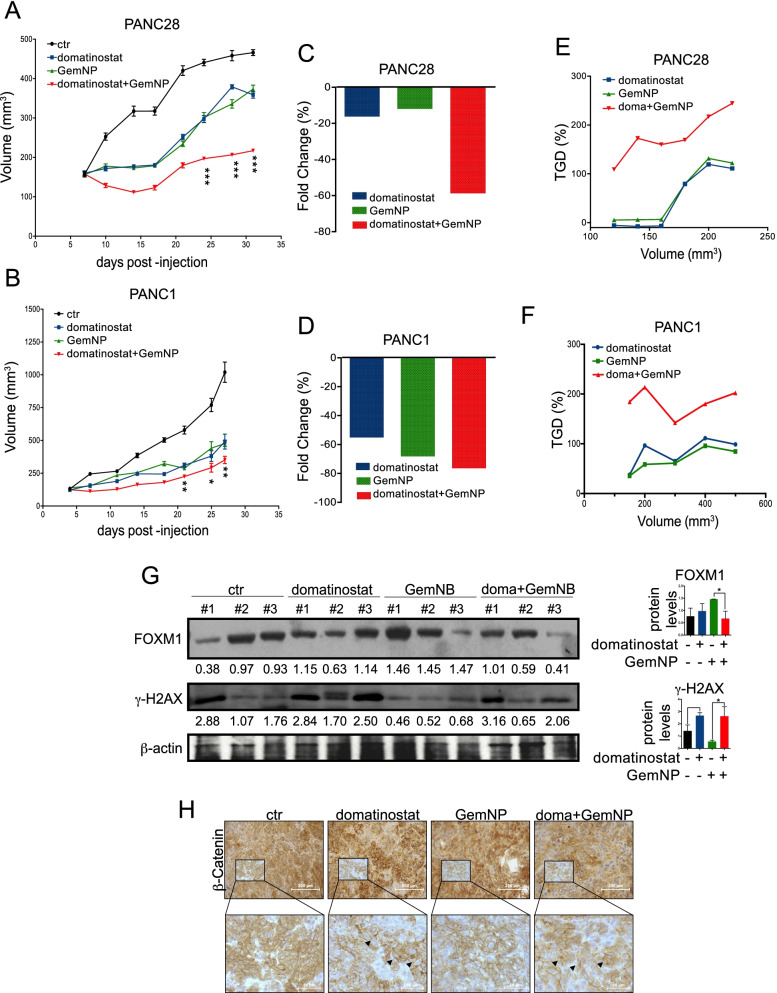
In vivo synergistic antitumor effect of domatinostat in combination with Gemcitabine plus Abraxane in PANC28 and PANC1 xenograft models. **A-B.** PANC28 and PANC1 cells were s.c. injected into athymic mice as described in the Materials and Methods. When established tumors were palpable, mice were treated with vehicles or domatinostat (20 mg/Kg 5 days/week, per os) alone and in combination with gemcitabine (weekly 25 mg/Kg, i.p.) and abraxane (weekly 20 mg/Kg, i.p.) (GT) for two weeks. Relative tumor volume curves are reported as mean ± SEM tumor volume measured at the indicated timing. **C-D.** Percent change in tumor volume average from first day of treatment (day 0) to the end of the study (day 32 for PANC28 xenograft and day 25 for PANC1 xenograft) for each treatment group compared to vehicles group (middle panels). **E-F.** Tumor growth delay (TGD), determined, in both PANC28 and PANC1 xenografts, as %TGD = [(T − C) /C] × 100, where T and C are the mean times expressed in days for the treated or control groups, respectively, to reach a defined tumor volume (see Materials and Methods). **G.** Expression of FOXM1 and γH2AX protein levels in lysates from three PANC1 xenograft tumor samples from each treatment group evaluated by WB. β-actin serves as control for equal protein loading. On the right, proteins quantification reported as mean ± SD for each treatment groups. **H.** Paraffin-embedded tissues were generated for each group for IHC analysis for β-Catenin as described in the Materials and Methods. Images were captured with a 20X (white scale bar: 200 μm) and 40X (white scale bar: 50 μm) objectives on a light microscope. Statistically significant results, obtained by 2-way ANOVA test, are reported (*** indicates *P* < 0.0005, ** indicates *P* < 0.005 and * indicates *P* < 0.05)

In order to confirm in vivo the synergistic antitumor effect observed in vitro we evaluated the activity of domatinostat in combination with chemotherapy in in PANC1 and PANC28 cell line xenograft mouse models. In detail, we evaluated combination treatment of domatinostat plus gemcitabine/nab-paclitaxel (GemNP) doublet, demonstrating in both PDAC models statistical significant decrease of tumor volume compared with control or single treatments (the treatment schedule is reported in Suppl. Fig. S13A) (Fig. 6A-B). Furthermore, by calculating the percent change in tumor volume from the time of initial treatment (day 0) to the end of the study (day 32 in PANC28 xenograft model and day 25 in PANC1 xenograft model), we confirmed that domatinostat plus GemNP combination significantly reduced the tumor burden in both models with a synergistic antitumor effect, compared to domatinostat or GemNP alone, particularly evident in PANC28 xenograft model (Fig. 6C-D).

The synergistic antitumor interaction was also tested by evaluating the tumor growth delay (TGD) induced by domatinostat plus GemNP, that reached a peak of more than 100%, indicating that the mean rate of tumor growth in the control was more than 3-fold higher than in the combination in both models (Fig. 6E-F). Notably, the maintenance of body weights (Suppl. Fig. S13 B-C) and the absence of other acute or delayed toxicity signs indicated a well tolerability of triple drugs combination.

We also validated in vivo the mechanistic findings evidenced in vitro on both FOXM1 modulation and CSCs targeting*.* In details, we found that the increased FOXM1 protein tumor expression observed in GemNP-treated mice was completely abolished by the concomitant treatment with domatinostat (Fig. 6G). Moreover, in line with in vitro data, FOXM1 down-modulation was paralleled also in vivo by a strong increase of γ-H2AX protein levels in both domatinostat and triple combination treatment group, indicating the induction of DNA damage (Fig. 6G).

Furthermore, we also found differential expression of β-Catenin in different treatment groups, with a prevalent membrane localization in domatinostat and triple combination, confirming in vivo the inhibition of Wnt-pathway by domatinostat (Fig. 6H).

Finally, although not statistically significant, we showed, also in vivo*,* a tendency of both FOXM1 and of CSC marker Oct-4 mRNA expression downregulation in xenograft tumors, induced by domatinostat alone or in combination with GemNP (Suppl. Fig. S14A and B).

**Fig. 7:**
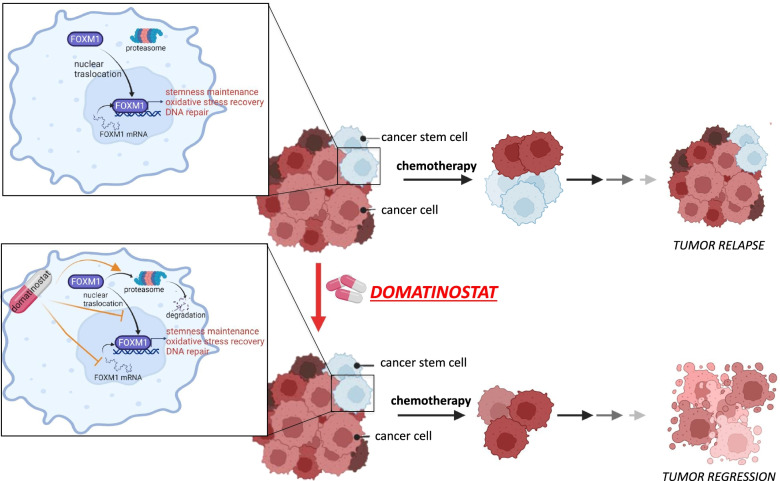
Graphical model. Domatinostat by preventing nuclear translocation and down-modulating FOXM1 expression, targets cancer stem cell compartment thus sensitizing PDAC cells to gemcitabine/nab-paclitaxel treatment

All together, these data confirmed the potential of a treatment strategy based on the addition of domatinostat to standard chemotherapy regimen to improve pancreatic cancer patient’s outcome and to bypass chemoresistance mechanisms. Moreover, these results further confirm our hypothesis that domatinostat potentiates chemotherapy in PDAC by targeting CSCs compartment via FOXM1 modulation (Fig. 7).

## Discussion

Here we showed that the novel HDACi domatinostat sensitizes PDAC cells, both in vitro in and in vivo xenograft models, to standard chemotherapeutics, including gemcitabine/nab-paclitaxel or fluoropyrimidine/irinotecan doublets, commonly used in PDAC patient treatment. Although a number of studies have already reported that domatinostat exerts antitumor effects in different tumor models [17, 24–27], our observation is the first to demonstrate synergistic interaction of domantinostat in combination setting with chemotherapy. We observed that simultaneous or sequential exposure of domatinostat, even at low doses, combined with GT doublet, resulted in synergistic anti-proliferative and pro-apoptotic effects related to a significant reduction of clonogenicity and PDAC spheroids viability, suggesting a mechanism involving the targeting of CSC compartment, a small subset of cancer cells displaying extremely resistance to conventional chemotherapy. Indeed, we showed that domatinostat decrease stem cell like features in PDAC cells both in vitro and in vivo, in line with previously reported effects in cancer cells, including PDAC [16, 17, 30]. However, we unveil a novel mechanism of domatinostat antitumor effect based on the modulation of CSC-redox homeostasis through the inhibition of the FOXM1 oncogene activity. We clearly showed that domatinostat induced in CSC subpopulation intracellular ROS accumulation particularly in mitochondrial compartment, directly connected with domatinostat-induced apoptotic effect. Moreover, we demonstrated that domatinostat, alone or in combination with GT, down-regulates FOXM1 mRNA and protein levels, particularly in CSC compartment, and prevents FOXM1 nuclear translocation and transcription activity, thus altering the expression of genes regulating redox homeostasis, DNA repair and stemness. These effects were also observed in PDAC in vivo models and confirmed by a network analysis further highlighting a functional relationship between FOXM1 and its target genes reported above. Most importantly, by generating FOXM1 PDAC overexpressing cells, we confirmed the critical role of the downregulation of this oncogene in domatinostat-induced sensitization to chemotherapy through ROS accumulation and CSC targeting.

Overexpression of FOXM1 has been detected in a broad range of cancer types, including PDAC, contributing to all hallmarks of cancer [12, 31, 32]. Moreover, FOXM1 regulatory network was recently suggested as a major predictor of adverse outcomes across several human malignancies [33]. In our study, by analyzing TCGA data we demonstrated a positive expression pattern correlation between FOXM1 and the its target genes regulating redox homeostasis, DNA repair and stemness in PDAC tissues. Furthermore, we also highlighted the correlation of FOXM1 high levels with both PFS and OS as well as with chemotherapy response in PDAC patients.

Although the role of FOXM1 in pancreatic cancer chemoresistance has not been explored in detail, previous studies in other tumor types suggested that FOXM1 can promote resistance by removing ROS, enhancing DNA damage repair and influencing tumor stemness [12]. Notably, in line with our findings, two independent groups demonstrated in gastric and colon rectal cancers that CSC have developed mechanisms for quenching excess ROS to maintain redox homeostasis including FOXM1-dependent Prx3 expression [34]. It was also reported that RAS plays a critical role in FOXM1 induction in cancer cells by ROS involvement [35]. However several different additional oncogenic stimuli/pathways including stemness pathways such as Hippo, Wnt, Hedgehog, were reported to affect expression and function of FOXM1 [36]. We added new insight in this mechanisms suggesting that CSC are addicted to FOXM1 overexpression because their high mitochondria ROS levels and oxidative stress adaptation. Interestingly, we and others have previously reported that one of the mechanisms of the antitumor effect exerted by HDACi is through the modulation of redox homeostasis [37]. We also recently highlighted the role of HDACi in targeting CSC compartment as a rationale for novel combinatorial approach with these agents to improve anticancer therapeutic efficacy and to revert drug resistance in solid tumors [14, 38].

The downregulation of FOXM1 mRNA expression was previously reported in atypical teratoid/rhabdoidtreated cancer by domatinostat [27] and similarly in hepatocellular carcinoma by the pan-HDACi vorinostat [39]. However, in our study we presented evidence demonstrating that mRNA down-modulation was also paralleled by a proteasome-dependent degradation of the protein induced by domatinostat and occurring within 6 h from treatment, both contributing to FOXM1 protein levels reduction and function hampering.

Several studies showed that FOXM1 levels can be altered in tumor cells by protein degradation regulated by ubiquitination and deubiquitination process [40]. On this regard the ubiquitin-specific protease 5 (USP5) has been recently associated with pancreatic cancer tumorigenesis and progression for its role in extending the half-life of FOXM1 by reducing its endogenous ubiquitination [29].

It was also reported that the aberrant activation of Wnt/β-catenin pathway, which is widespread in human cancers, including pancreatic cancer [14], favors the interaction between FOXM1 and USP5, thereby inducing FOXM1 protein stabilization and nuclear accumulation in glioma cells [13]. In turn FOXM1 in the nucleus recruits β-catenin to Wnt target-genes representing an additional mechanism for controlling canonical Wnt signaling and cancer cell proliferation. Notably Wnt signaling pathway is one of the major morphogenic pathways in stem cells playing a critical role in CSC regulation [14].

Indeed, in our study we showed that the high FOXM1 expression in CSC enriched PDAC spheroids was accompanied by increased β-catenin expression, compared to differentiated cells. Most interestingly inhibition of FOXM1 nuclear translocation induced by domatinostat was paralleled and even preceded by that of β-catenin. Furthermore, we showed an induction of both FOXM1 and β-catenin upon chemotherapy treatment that was completely abolished by concomitant treatment with domatinostat and paralleled by increased DNA-damage in combination setting, both in vitro and in vivo models. Overall, we can speculate that domatinostat induces a Wnt-pathway repression that leads to a FOXM1 retain in the cytoplasm in the early time frame, followed by a transcriptional down-regulation. On this regard, some evidence demonstrated that HDAC inhibition led to a decrease in β-catenin nuclear localization, resulting in a strong inhibition of cell proliferation [41, 42].

However, additional mechanistic studies, at the moment not within the scope of the present study, should be performed in order to confirm this hypothesis.

Domatinostat, currently in clinical development in both in hematological and solid malignancies, has shown a good safety profile also in combination treatments [43–46]. In this regard, in our study we demonstrated a selective anti-tumour effect of domatinostat on tumor cells and a good tolerability of treatment in combination with chemotherapy in mice preclinical model.

## Conclusion

In summary, our findings provide several evidence supporting the idea that class I HDAC inhibitors such as domatinostat, by acting on CSC compartment via modulation of FOXM1-mediated mitochondrial and cellular homeostasis, could improve the efficacy and overcome the resistance of commonly used chemotherapeutics in PDAC cells (Fig. 7). Therefore, we suggest a novel combination therapeutic strategy based on domatinostat against metastatic PDAC, a malignancy with very poor prognosis, that warrant further clinical evaluation. Our mechanistic insight could suggest a stratification strategy based on FOXM1 expression to select patients that could benefit of combination treatment.

## Supplementary Information

**Additional file 1: Supplementary Table S1.** Pancreatic cancer cells sensitivity. **Supplementary Table S2.** Screening domatinostat in combination with chemotherapeutics on cell proliferation by SRB assay and Calcusyn software. **Supplementary Table S3.** Screening domatinostat in combination with chemotherapeutics on cell proliferation by SRB assay and Calcusyn software. **Supplementary Table S4.** Screening domatinostat in combination with chemotherapeutics on cell proliferation by SRB assay and Calcusyn software. **Supplementary Table S5.** Screening domatinostat in combination with chemotherapeutics on cell proliferation by SRB assay and Calcusyn software. **Supplementary Table S6.** Domatinostat modulates FOXM1 activity. **Supplementary Table S7.** Critical FOXM1 role in pancreatic cancer patients. **Supplementary Figure S1.** Chemotherapy effect on pancreatic cancer cells. **Supplementary Figure S2**. Domatinostat plus gemcitabine/taxol combination induces Annexin-V exposure. **Supplementary Figure S3.** Domatinostat plus gemcitabine/taxol combination induces cell cycle perturbation and a clear block in phase S. **Supplementary Figure S4.** Pancreatic cancer cells enriched in stem-cell features, culturing model validation. **Supplementary Figure S5.** Domatinostat effects on CSC population. **Supplementary Figure S6.** The effect of domatinostat on PDAC spheroid cultures. **Supplementary Figure S7.** Domatinostat effects on FOXM1 expression. **Supplementary Figure S8.** Domatinostat modulates FOXM1 activity. **Supplementary Figure S9.** A network analysis revealed FOXM1 as upstream regulator of significant domatinostat-modulated proteins involved in stemness, oxidative stress and DNA repair. **Supplementary Figure S10.** Critical FOXM1 role in pancreatic cancer patients. **Supplementary Figure S11.** FOXM1 expression in transfected cells. **Supplementary Figure S12.** Domatinostat modulates FOXM1 activity. **Supplementary Figure S13.** Domatinostat does not exert toxic effect alone and in combination in vivo. **Supplementary Figure S14.** Domatinostat synergistically improves overall survival of PANC1 mice affecting FOXM1 and OCT4 expression.

## Data Availability

All data generated or analyzed during this study are included in this published article or in supplementary information. Materials, additional data, and protocols described in the manuscript will be made available from the corresponding authors upon reasonable request. Raw data are available at 10.6084/m9.figshare.19368845

## Abbreviations

PDAC: Pancreatic ductal adenocarcinoma
CSC: Cancer stem cells
HDACi: Histone deacetylase inhibitors
5’DFUR: 5′-deoxy-5-fluoro-uridine
CI: Combination index
DRI: Dose reduction index
GT: Gemcitabine/taxol
BJhTERT: hTERT-immortalized foreskin fibroblast
PARP: PolyADP-ribose polymerase
OCT4 or Oct-4: octamer-binding transcription factor 4
ROS: Reactive oxygen species
NAC: N-acetylcysteine
IF: Immunofluorescence
FOXM1: Forkhead box protein M1
TCGA: The Cancer Genome Atlas
WB: Western Blot
CAT: Catalase
SOD2: Superoxide dismutase
GPX2: Glutathione peroxidase 2
RAD51: RAD51 Recombinase
XRCC1: X-ray repair cross complementing 1
SOX2: SRY-Box Transcription Factor 2
BIRC5: Baculoviral IAP Repeat Containing 5
ChIP: Chromatine-immunoprecipitation
OE-FOXM1: FOXM1 over-expressing
EV-FOXM1: FOXM1 empty vector
GemNP: gemcitabine/nab-paclitaxel
TGD: Tumor growth delay
IHC: Immunohistochemistry
