# Correction to: HDAC class I inhibitor domatinostat sensitizes pancreatic cancer to chemotherapy by targeting cancer stem cell compartment via FOXM1 modulation

**DOI:** 10.1186/s13046-022-02324-2

**Published:** 2022-04-11

**Authors:** Maria Serena Roca, Tania Moccia, Federica Iannelli, Cristina Testa, Carlo Vitagliano, Michele Minopoli, Rosa Camerlingo, Giulia De Riso, Rossella De Cecio, Francesca Bruzzese, Mariarosaria Conte, Lucia Altucci, Elena Di Gennaro, Antonio Avallone, Alessandra Leone, Alfredo Budillon

**Affiliations:** 1 Experimental Pharmacology Unit-Laboratory of Naples and Mercogliano (AV), Istituto Nazionale per lo Studio e la Cura dei Tumori “Fondazione G. Pascale” – IRCCS, Naples, Italy; 2 Neoplastic Progression Unit, Istituto Nazionale per lo Studio e la Cura dei Tumori “Fondazione G. Pascale” – IRCCS, Naples, Italy; 3 Cell Biology and Biotherapy Unit, Istituto Nazionale per lo Studio e la Cura dei Tumori “Fondazione G. Pascale” – IRCCS, Naples, Italy; 4 Department of Molecular Medicine and Medical Biotechnology, University of Naples “Federico II”, Naples, Italy; 5 Pathology Unit, Istituto Nazionale per lo Studio e la Cura dei Tumori “Fondazione G. Pascale” – IRCCS, Naples, Italy; 6 Animal Facility, Istituto Nazionale per lo Studio e la Cura dei Tumori “Fondazione G. Pascale” – IRCCS, Naples, Italy; 7 Department of Precision Medicine, Universita degli Studi della Campania “Luigi Vanvitelli”, Naples, Italy; 8 BIOGEM, (AV), Naples, Italy; 9 Experimental Clinical Abdominal Oncology, Istituto Nazionale per lo Studio e la Cura dei Tumori “Fondazione G. Pascale” – IRCCS, Naples, Italy

**Correction to: J Exp Clin Cancer Res 41, 83 (2022)**

**https://doi.org/10.1186/s13046-022-02295-4**

Following publication of the original article [1], the authors identified minor errors in Figs. 3 and 5; specifically:

- Fig. 3e: in the upper panel, the standard deviation lines and statistically lines originally overlapped; this has been corrected
- Fig. 5n: the western blot for FOXM1 originally contained an erroneous ‘16h’ notation on the band; this has been removed

The corrected figures are given here.

**Fig. 3:**
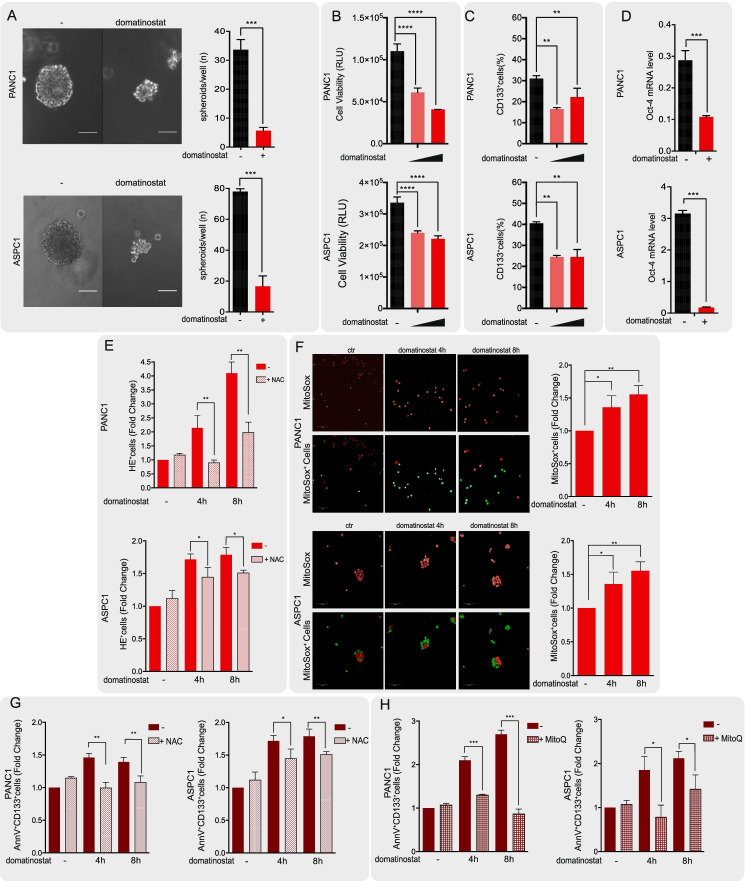
Domatinostat affects PDAC stem cells by modulating oxidative stress. **A** The effect of domatinostat (0,5 μM) on PANC1 and ASPC1 spheroid cultures. Cells (1000/mL) seeded in a matrigel drop and sphere medium, were treated with and without domatinostat and collected 7 days after treatment. Images of one spheroid for each condition in a representative experiment is shown (white scale bar: 50 μm, magnification 20X). On the right, bar graphs show the numbers of spheroids for well (mean ±} SD of 2 or more separate experiments each one with technical triplicate). **B** PANC1 and ASPC1 spheroids viability treated with and without domatinostat (0.5 μM and 1 μM) was assessed by cell titer luminescence assay (see Materials and Methods) (mean ±} SD of 2 or more separate experiments each one with technical triplicate). **C** Flow cytometry assay shows CD133 protein expression decrease after domatinostat (0.5 and 1 μM) treatment for 16 h in PANC1 and ASPC1 cells. **D** qRT-PCR analysis shows Oct-4 levels drop when PANC1, and ASPC1 spheroids are treated with domatinostat (0.5 μM) for 16 h. **E** Cellular ROS production is visualized by Hydroethidine (HE) staining. PANC1 and ASPC1 spheroids were treated with domatinostat (0.5 μM) alone and in combination with N-acetylcysteine (NAC, 5 mM), as ROS scavenger, at the indicated timing. Cells were stained for HE as described in Material and Methods section and visualized by flow cytometry. **F** Mitochondrial ROS amount is analyzed by mitosox staining. PANC1 and ASPC1 spheroids treated with or without domatinostat (0.5 μM) alone at the indicated timing were fixed, stained for mitosox (red) and measured by Opera Phenix confocal microscopy. The mitosox positive cells are counted by Harmony software as described in Material and Methods section. Representative images (20X magnification) show stained cells (red) and mitosox counted positive cells (green). **G-H** The observed increase in ROS amount is related to an increase of apoptotic cancer stem cells upon domatinostat treatment. PANC1 and ASPC1 spheroids, treated as previously, were stained for AnnexinV-FITC and CD133-APC as described in Material and Methods section and visualized by flow cytometry. In G. PANC1 and ASPC1 spheroids were treated with domatinostat (0.5 μM) alone and in combination with NAC, 5 mM, as ROS scavenger, at the indicated timing. In **H** PANC1 and ASPC1 spheroids were treated with domatinostat (0.5 μM) alone and in combination with Mitoquinone mesylate (MitoQ) 100 nM, as mitochondrial ROS scavenger, at the indicated timing. (Statistically significant results by ANOVA test are reported *** indicates *P* < 0.0001, ** indicates *P* < 0.005 and * indicates *P* < 0.05)

**Fig. 5:**
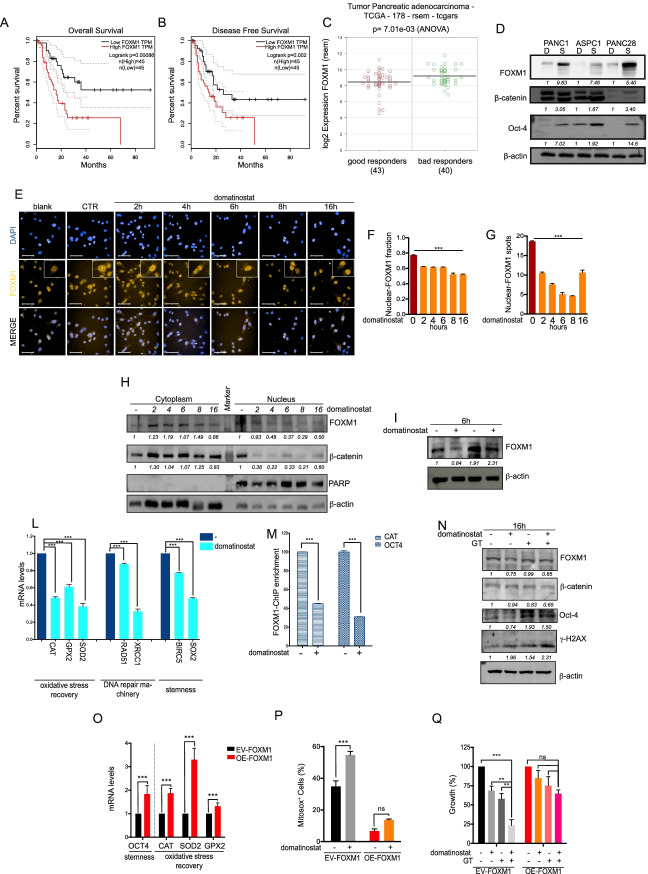
Domatinostat potentiates chemotherapy effect by modulating expression and localization of FOXM1 in CSCs. **A** FOXM1 expression in patients with poor (dead; *n* = 93) and good prognosis (alive; *n* = 85) in the TCGA PAAD cohort (Wilcox-test W = 2647, *p*-value< 0.00058). **B** FOXM1 expression in sensitive and resistant to the primary therapy in the TCGA PAAD cohort, evaluated as PFS (Wilcox-test W = 2269, *p*-value< 0.00012). **C** FOXM1 expression in good (complete-remission_response patients, *n* = 43) vs bad (progressive-disease patients, *n* = 40) responders to chemotherapy, picked in “treatment_outcome_first_course” subset patients (One Way Analysis of variance; *p*-value< 0.000701). **D** Basal protein levels of FOXM1 and stem-cell markers (β-Catenin; Oct-4) in PANC1, PANC28 and ASPC1 spheroids (S) versus differentiated cells (D). β-actin serves as loading protein control. **E** Nuclear localization by IF upon domatinostat (0.5 μM) treatment in PANC1 spheroid cells at indicated timing. Bar 50 μM. Magnification 40X. DAPI is for nuclear staining. **F** FOXM1-nuclear intensity quantified by Harmony software. **G**. FOXM1-nuclear spots were quantified by Harmony software. **H** WB analysis of nuclear and cytoplasmic FOXM1 in PANC1 spheroids treated with domatinostat (0.5 μM) at the indicated timing. PARP and β-actin serve as nuclear and cytoplasmic loading control, respectively. **I** FOXM1 protein expression in PANC1 spheroids, treated with domatinostat (0.5 μM) and domatinostat plus Bortezomib (20 mM) for 6 h. β-actin serves as loading control. **L** CAT, GPX2, SOD2, RAD51, XRCC1, BIRC5 and SOX2 mRNA levels in PANC1 spheroids, treated with domatinostat (0.5 μM) for 16 h. **M** ChIP-qPCR analysis showing the relative decrease of enrichment of FOXM1 binding to CAT and OCT4 promoters. Data obtained on immunoprecipitated fractions were normalized to input chromatin (IP/Input). The mean of at least two independent experiments with error bars indicating the SD. **N** FOXM1, β-Catenin, Oct-4 and γH2AX protein expression in PANC1 spheroids treated for 16 h with domatinostat, GT (IC_50_^96^h) or their combination. β-actin serves as loading control. **O** OCT4, CAT, SOD2 and GPX2 mRNA levels in PANC1 cells transfected with FOXM1 (OE-FOXM1) or empty vector (EV-FOXM1). **P** Mitochondrial ROS amount in OE-FOXM1 and EV-FOXM1 PANC1, treated or untreated with domatinostat (0.5 μM) for 16 h, visualized by mitosox staining. **Q** OE-FOXM1 and EV-FOXM1 PANC1 cells were treated for 96 h with domatinostat (1 μM) alone or in combination with GT (respectively, 100 nM and 1.56 nM). Cell growth expressed as percentage of control was assessed by SRB colorimetric assay. The values are the means ±}S.D. from at least three independent experiments. Statistically significant results, by 2-way ANOVA test, are reported (***indicates *P* < 0.0001, **indicates *P* < 0.005 and *indicates *P* < 0.05)

In the Reagents section of the Materials and Methods, the second sentence originally contained a typo. The antibody name was listed as ‘poly-(ADPribose)-Polymerase (PARP)-Ab (#556494)’; the ‘-Ab’ was included in error. The correct sentence therefore reads:

Primary antibodies for western blotting were used according to the manufacturer’s protocol: β-Actin (#8227), poly-(ADPribose)-Polymerase (PARP) (#556494), phospho-Histone H2AX (γH2AX) (#05636), FOXM1 (#5436S), β-catenin (#8480S), Oct-4 (#2750S), were purchased from Cell signaling Technology (Danvers, MA, USA). γ-Tubulin (#sc-7396) were purchased from Santa Cruz Biotechnology (Dallas, TX, USA).

In addition, in the caption for Fig. 6a-b, the description should read as follows (emphasis placed on adjusted wording, which originally said ‘(GT)’:

**A-B.** PANC28 and PANC1 cells were s.c. injected into athymic mice as described in the Materials and Methods. When established tumors were palpable, mice were treated with vehicles or domatinostat (20 mg/Kg 5 days/week, per os) alone and in combination with gemcitabine (weekly 25 mg/Kg, i.p.) and abraxane (weekly 20 mg/Kg, i.p.) **(GemNP)** for two weeks.

In the Availability of data and materials section, it has been updated to: All data generated or analyzed during this study are included in this published article or in supplementary information. Materials, additional data, and protocols described in the manuscript will be made available from the corresponding authors upon reasonable request. Raw data are available at 10.6084/m9.figshare.19368845.

The corrections do not have any effect on the final conclusions of the paper. The original article has been corrected.
