# Supplementary material for: HDAC class I inhibitor domatinostat sensitizes pancreatic cancer to chemotherapy by targeting cancer stem cell compartment via FOXM1 modulation
Source: J Exp Clin Cancer Res. 2022 Mar 3;41:83. doi: 10.1186/s13046-022-02295-4 (PMC8892808; doi:10.1186/s13046-022-02295-4)
Supplement: Supplementary file 1 — Additional file 1: Supplementary Table S1. Pancreatic cancer cells sensitivity. Supplementary Table S2. Screening domatinostat in combination with chemotherapeutics on cell proliferation by SRB assay and Calcusyn software. Supplementary Table S3. Screening domatinostat in combination with chemotherapeutics on cell proliferation by SRB assay and Calcusyn software. Supplementary Table S4. Screening domatinostat in combination with chemotherapeutics on cell proliferation by SRB assay and Calcusyn software. Supplementary Table S5. Screening domatinostat in combination with chemotherapeutics on cell proliferation by SRB assay and Calcusyn software. Supplementary Table S6. Domatinostat modulates FOXM1 activity. Supplementary Table S7. Critical FOXM1 role in pancreatic cancer patients. Supplementary Figure S1. Chemotherapy effect on pancreatic cancer cells. Supplementary Figure S2. Domatinostat plus gemcitabine/taxol combination induces Annexin-V exposure. Supplementary Figure S3. Domatinostat plus gemcitabine/taxol combination induces cell cycle perturbation and a clear block in phase S. Supplementary Figure S4. Pancreatic cancer cells enriched in stem-cell features, culturing model validation. Supplementary Figure S5. Domatinostat effects on CSC population. Supplementary Figure S6. The effect of domatinostat on PDAC spheroid cultures. Supplementary Figure S7. Domatinostat effects on FOXM1 expression. Supplementary Figure S8. Domatinostat modulates FOXM1 activity. Supplementary Figure S9. A network analysis revealed FOXM1 as upstream regulator of significant domatinostat-modulated proteins involved in stemness, oxidative stress and DNA repair. Supplementary Figure S10. Critical FOXM1 role in pancreatic cancer patients. Supplementary Figure S11. FOXM1 expression in transfected cells. Supplementary Figure S12. Domatinostat modulates FOXM1 activity. Supplementary Figure S13. Domatinostat does not exert toxic effect alone and in combination in vivo. Supplementary Figure S14. [file 13046_2022_2295_MOESM1_ESM.pdf]

IC50 96h						
Cell lines	*domatinostat µM*	*Gemcitabine nM*	*Taxol nM*	*5' DFUR µM*	*SN-38 nM*	*Oxaliplatin µM*
**PANC1**	0.56 ± 0.19	16.72 ± 2.05	6.75 ± 1.06	26.68 ± 8.51	3.33 ± 1.46	1.30 ±0.42
**ASPC1**	0.47 ± 0.14	13.87 ± 2.42	3.69 ± 1.80	30.00 ± 8.46	25.58 ± 5.16	26.29 ±4.16
**PANC28**	0.32 ± 0.03	54.20 ± 6.14	1.41 ± 0.50	10.30 ± 4.53	5.38 ± 1.72	2.07 ±1.60
**BJhTERT**	0.66 ± 0.28	ND	49 ± 1.41	56.7± 5.7	27.5 ± 3.53	8.2 ± 1.87

Supplementary Table S1. **Pancreatic cancer cells sensitivity.**
Anti-proliferative effects of domatinostat and gemcitabine, taxol, 5'DFUR, SN-38 and oxaliplatin evaluated as half maximal inhibitory concentration (IC50) upon 96 hours of treatment.

Cell lines	treatment	50:50 Combination domatinostat + Chemio 96h				50:50 Sequential Treatment domatinostat 96h -> Chemio 72h			
		CI_{50} (±SD)	CI_{75} (±SD)	DRI_{50} (±SD) domatinostat	DRI at CI_{50} (±SD) chemio	CI_{50} (±SD)	CI_{75} (±SD)	DRI_{50} (±SD) domatinostat	DRI at CI_{50} (±SD) chemio
PANC28	domatinostat+Gemcitabine (Gem)	0.90±0.12	0.89±0.03	1.52±0.14	3.35±1.15	0.84±0.06	0.64±0.21	2.10±0.00	2.98±0.64
	domatinostat+Taxol (Tax)	0.50±0.12	0.44±0.09	15.15±13.64	16.17±12.85	0.66±0.26	0.75±0.32	3.17±0.14	3.57±2.16
	domatinostat+GemTax	0.92±0.16	1.11±0.15	1.33±0.75	1.37±0.31	1.08±0.02	1.03±0.42	1.56±0.26	2.35±0.43
PANC1	domatinostat+Gemcitabine (Gem)	1.05±0.13	1.07±0.16	2.23±0.6	1.4±0.18	0.75±0.07	0.60±0.29	2.12±0.38	4.88±0.25
	domatinostat+Taxol (Tax)	0.67±0.14	0.60±0.11	2.36±0.72	4.22±2.65	0.60±0.07	0.75±0.35	3.30±1.92	2.86±1.60
	domatinostat+GemTax	0.52±0.35	0.84±0.1	2.9±1.3	1.98±0.66	1.03±0.16	0.89±0.14	1.60±0.45	1.82±0.26
ASPC1	domatinostat+Gemcitabine (Gem)	0.96±0.80	0.90±0.18	4.36±2.86	3.57±2.18	0.82±0.03	0.55±0.13	1.85±0.35	3.70±0.56
	domatinostat+Taxol (Tax)	0.74±0.24	1.07±0.55	2.71±1.68	4.09±0.99	0.81±0.33	1.59±0.6	3.29±1.44	1.63±0.38
	domatinostat+GemTax	0.86±0.02	1.01±0.16	3.15±1.34	3.43±0.32	1.27±0.13	1.11±0.06	1.55±0.47	1.58±1.11
BJhTERT	domatinostat+Gemcitabine (Gem)	ND	ND	ND	ND	ND	ND	ND	ND
	domatinostat+Taxol (Tax)	2.51±0.66	12.14±17.28	0.56±0.35	7.16±7.71	1.28±0.58	1.4±1.12	3.54±2.51	2.15±1.9
	domatinostat+GemTax	ND	ND	ND	ND	ND	ND	ND	ND

< 0,9 synergism
0,9< x <1,10 additivity

Supplementary Table 2. Screening domatinostat in combination with chemotherapeutics on cell proliferation by SRB assay and Calcusyn software. Combination Index 50 and Index 75 of simultaneous and sequential treatment of domatinostat plus Chemiotherapeutics (gemcitabine, taxol and gemcitabine plus taxol) at equi-toxic doses .

		75:25 Combination domatinostat+ Chemio 96h				75:25 Sequential Treatment domatinostat 96h -> Chemio 72h			
Cell lines	*treatment*	*CI_{50} (±SD)*	*CI_{75} (±SD)*	*DRI_{50} (±SD) domatinostat*	*DRI at CI_{50} (±SD) chemio*	*CI_{50} (±SD)*	*CI_{75} (±SD)*	*DRI_{50} (±SD) domatinostat*	*DRI at CI_{50} (±SD) chemio*
PANC28	domatinostat+Gemcitabine (Gem)	0.71±0.32	0.90±0.5	1.24±0.57	7.03±2.41	0.66±0.15	0.64±0.16	1.57±0.41	10.04±3.20
	domatinostat+Taxol (Tax)	0.74±0.16	0.58±0.21	8.51±1.81	1.60±0.77	0.47±0.35	0.66±0.35	1.38±0.28	10.29±5.82
	domatinostat+GemTax	0.80±0.11	0.84±0.12	1.52±0.44	6.27±3.11	1.04±0.08	0.74±0.06	1.18±0.20	3.38±1.69
PANC1	domatinostat+Gemcitabine (Gem)	0.46±0.03	0.71±0.12	10.18±5.03	2.22±1.54	0.65±0.12	0.68±0.16	1.56±0.30	21.14±3.37
	domatinostat+Taxol (Tax)	0.67±0.04	0.87±0.24	1.56±0.58	4.09±0.99	1.17±0.22	1.17±0.36	0.99±0.29	4.2±2
	domatinostat+GemTax	0.58±0.11	0.78±0.03	1.8±0.45	8.3±1.41	0.73±0.23	0.7±0.02	2.12±1.30	6.35±2.00
ASPC1	domatinostat+Gemcitabine (Gem)	0.50±0.27	0.56±0.21	2.57±1.22	2.52±1.22	0.66±0.08	0.65±0.21	1.55±0.21	26.30±3.25
	domatinostat+Taxol (Tax)	0.67±0.04	1.05±0.28	1.17±0.23	4.09±0.99	0.68±0.14	0.85±0.32	1.69±0.28	8.83±2.47
	domatinostat+GemTax	0.79±0.08	0.90±0.05	1.39±0.09	7.07±1.46	0.72±0.18	1.04±0.03	1.50±0.48	17.43±2.31
BJhTERT	domatinostat+Gemcitabine (Gem)	ND	ND	ND	ND	ND	ND	ND	ND
	domatinostat+Taxol (Tax)	1.85±1.7	1.65±1.13	0.91±0.67	48.45±13.05	1.1±0.19	1.20±0.12	2.4±0.	1.46±0.61
	domatinostat+GemTax	ND	ND	ND	ND	ND	ND	ND	ND

< 0,9 synergism

0,9< x <1,10 additivity

Supplementary Table 3. Screening domatinostat in combination with chemotherapeutics on cell proliferation by SRB assay and Calcusyn software. Combination Index 50 and Index 75 of simultaneous and sequential treatment of domatinostat plus Chemiotherapeutics (gemcitabine, taxol and gemcitabine plus taxol) with domatinostat excess dose (75:25).

		50:50 Combination domatinostat+ Chemio 96h				50:50 Sequential Treatment domatinostat 96h ->Chemio 72h			
Cell lines	*treatment*	*CI_{50} (±SD)*	*CI_{75} (±SD)*	*DRI_{50} (±SD) domatinostat*	*DRI at CI_{50} (±SD) chemio*	*CI_{50} (±SD)*	*CI_{75} (±SD)*	*DRI_{50} (±SD) domatinostat*	*DRI at CI_{50} (±SD) chemio*
PANC28	domatinostat+5'DFUR	1.06±0.03	1.12±0.10	2.73±0.40	1.42±0.03	0.62±0.25	0.56±0.27	245±1.06	2.66±0.19
	domatinostat+ *SN-38*	0.68±0.14	0.58±0.29	1.23±0.22	1.23±0.22	0.65±0.07	0.91±0.27	1.62±0.86	2.18±2.00
	domatinostat+ 5'DFUR/*SN-38*	1.22±0.08	0.90±0.27	2.94±0.82	2.88±0.11	0.89±0.16	0.89±0.01	2.14±0.57	2.83±1.24
	domatinostat+Oxaliplatin	0.82±0.39	0.91±0.37	2.5±1.13	3.1±1.41	0.75±0.17	0.68±0.04	2.19±1.00	2.18±0.29
PANC1	domatinostat+5'DFUR	1.05±0.32	1.36±0.26	2.73±0.06	1.78±1.03	0.47±0.00	0.48±0.06	2.97±0.16	6.82±0.55
	domatinostat+ *SN-38*	1.08±0.03	0.80±0.13	7.55±1.2	2.4±0.42	0.60±0.29	0.76±0.29	1.29±0.60	2.15±1.26
	domatinostat+ 5'DFUR/*SN-38*	1.10±0.13	1.03±0.02	2.19±0.98	1.46±0.79	0.65±0.16	0.53±0.09	5.95±2.05	2.72±0.44
	domatinostat+Oxaliplatin	0.79±0.25	0.77±0.27	2.32±1.02	3.45±1.31	0.67±0.07	0.85±0.15	2.96±1.81	2.83±1.56
ASPC1	domatinostat+5'DFUR	1.05±0.06	2.54±0.36	2.36±0.08	1.46±0.80	0.88±0.09	0.88±0.13	1.64±0.09	38.00±0.00
	domatinostat+ *SN-38*	0.45±0.21	0.81±0.09	3.35±1.81	1.87±0.84	0.73±0.06	0.98±0.16	1.68±1.15	61.46±18.75
	domatinostat+ 5'DFUR/*SN-38*	0.75±0.08	0.70±0.15	2.02±1.07	2.90±1.40	0.66±0.02	0.93±0.03	1.41±0.49	6.35±2.94
	domatinostat+Oxaliplatin	0.81±0.03	0.76±0.02	2.19±0.38	2.88±0.96	0.47±0.05	0.81±0.39	3.42±1.01	8.69±7.08
BJhTERT	domatinostat+5'DFUR	0.74±0.41	1.02±0.23	1.87±1.16	5.46±5.08	1.18±0.28	1.57±0.42	1.60±0.26	3.91±1.13
	domatinostat+ *SN-38*	0.89±0.25	3.93±1.52	2.08±10.7	2.92±0.62	1.00±0.12	1.23±0.5	1.5±0.14	3.43±1.86
	domatinostat+ 5'DFUR/*SN-38*	1.25±0.18	1.96±0.34	1.69±0.75	1.75±0.24	1.06±0.10	1.22±0.18	1.65±0.54	3.33±1.57
	domatinostat+Oxaliplatin	1.05±0.15	1.43±0.35	1.70±0.71	2.83±1.04	1.02±0.04	1.17±0.29	1.90±0.70	3.01±1.21

- < 0,9 synergism
- 0,9< x <1,10 additivity

Supplementary Table 4. Screening domatinostat in combination with chemotherapeutics on cell proliferation by SRB assay and Calcusyn software. Combination Index 50 and Index 75 of simultaneous and sequential treatment of domatinostat plus chemotherapeutics (5'DFUR, SN-38, oxaliplatin and 5'DFUR plus SN-38) at equitoxic doses (50:50). **B.** Combination Index 50 and Index 75 of simultaneous and sequential treatment of domatinostat plus chemotherapeutics (5'DFUR, SN-38, oxaliplatin and 5'DFUR plus SN-38) with an excess of domatinostat (75:25).

		75:25 Combination domatinostat+ Chemio 96h				75:25 Sequential Treatment domatinostat 96h ->Chemio 72h			
Cell lines	*treatment*	*CI_{50} (±SD)*	*CI_{75} (±SD)*	*DRI_{50} (±SD) domatinostat*	*DRI at CI_{50} (±SD) chemio*	*CI_{50} (±SD)*	*CI_{75} (±SD)*	*DRI_{50} (±SD) domatinostat*	*DRI at CI_{50} (±SD) chemio*
PANC28	domatinostat+5'DFUR	0.95±0.05	0.71±0.17	1.41±0.30	2.58±0.55	0.64±0.19	0.61±0.20	2.0±0.56	7.97±2.58
	domatinostat+ SN3-8	0.81±0.14	1.09±0.04	1.23±0.20	4.80±2.60	0.56±0.32	0.68±0.19	1.75±1.19	24.92±5.14
	domatinostat+ 5'DFUR/SN-38	0.61±0.09	0.96±0.08	2.16±0.50	5.30±1.69	0.59±0.07	0.63±0.14	1.52±0.39	29.8±6.95
	domatinostat+Oxaliplatin	0.70±0.36	0.92±0.36	1.69±1.05	3.12±0.43	1.03±0.04	1.02±0.02	1.07±0.30	3.75±0.45
PANC1	domatinostat+5'DFUR	0.87±0.07	1.10±0.54	1.24±0.27	4.29±0.3.52	0.61±0.02	0.61±0.20	2±0.56	7.97±2.58
	domatinostat+ SN-38	1.18±0.14	1.26±0.26	0.98±0.08	4.09±1.55	0.53±0.10	1.24±0.21	1.34±0.70	16.05±9.31
	domatinostat+ 5'DFUR/SN-38	1.07±0.11	1.45±0.51	1.14±0.38	2.62±1.05	0.86±0.03	0.66±0.04	1.59±0.40	7.63±2.87
	domatinostat+Oxaliplatin	0.77±0.103	0.52±0.24	3.23±1.16	2.66±0.87	1.47±0.50	1.08±0.05	0.79±0.23	3.71±0.19
ASPC1	domatinostat+5'DFUR	1.08±0.04	1.09±0.09	1.13±0.16	3.49±0.43	0.67±0.16	1.05±0.02	1.82±0.13	7.28±5.74
	domatinostat+ SN-38	1.29±0.12	1.63±0.61	0.85±0.01	37.8±12.3	0.86±0.23	0.98±0.11	1.54±0.48	9.61±1.68
	domatinostat+ 5'DFUR/SN-38	0.9±0.17	1.08±0.18	1.18±0.36	4.91±1.66	0.75±0.09	0.63±0.17	1.36±0.19	29.45±0.07
	domatinostat+Oxaliplatin	1.24±0.06	1.65±0.51	0.89±0.04	8.80±3.79	0.73±0.13	0.88±0.02	1.21±0.78	3.05±1.59
BJhTERT	domatinostat+5'DFUR	0.68±0.6	0.96±0.16	2.54±2.2	21.4±11.6	1.65±0.57	0.83±0.14	1.53±0.73	14.58 ±3.19
	domatinostat+ SN-38	2.76±1.47	2.45±0.62	0.44±.25	23.18±3.15	1.51±0.18	1.31±0.40	0.88±0.35	9.77±1.47
	domatinostat+ 5'DFUR/SN-38	1.88±0.70	1.42±0.52	1.11±0.53	3.45±0.53	1.26±0.19	1.02±0.15	1.53±0.57	14.58 ±3.19
	domatinostat+Oxaliplatin	1.14±0.07	1.4±0.38	1.12±0.31	4.25±2.05	0.85±0.12	1.25±0.55	1.34±0.21	10.67±3.36

< 0,9 synergism
0,9< x <1,10 additivity

Supplementary Table 5. Screening domatinostat in combination with chemotherapeutics on cell proliferation by SRB assay and Calcusyn software. Combination Index 50 and Index 75 of simultaneous and sequential treatment of domatinostat plus chemitherapeutics (5'DFUR, SN-38, oxaliplatin and 5'DFUR plus SN-38) with an excess of domatinostat (75:25).

Oxidative stress genes related to bad prognosis [9]	*baseMean*	*log_2FoldChange*	*lfcSE*	*stat*	*pvalue*	*padj*
**FOXM1**	1.33E+03	-3.79E+00	1.04E-01	-3.63E+01	3.37E-288	1.07E-285
**NUDT1**	1.49E+02	-2.25E+00	1.89E-01	-1.19E+01	1.74E-32	1.52E-31
**RNF7**	6.95E+02	-7.76E-02	1.02E-01	-7.61E-01	4.47E-01	5.09E-01
**EPHX2**	1.40E+01	-2.04E-02	4.97E-01	-4.11E-02	9.67E-01	9.74E-01
**SEPP1**	6.36E+01	2.04E+00	2.69E-01	7.61E+00	2.83E-14	1.16E-13
**DUSP1**	2.25E+03	2.20E+0	8.37E-02	2.63E+01	3.48E-152	3.16E-150
**TXNRD1**	1.52E+04	2.22E+00	6.50E-02	3.41E+01	1.58E-254	4.18E-252

Supplementary Table S6. **Domatinostat modulates FOXM1 activity.**
The top stress-related genes [9] that established the greatest fold change in pancreatic cancer cells when domatinostat is administered at low dose for 24 hours compared to DMSO treatment. The RNA-Seq data are obtained from a domatinostat transcriptome-wide effects study in L3.6, BxPC3 and PANC1, performed by Mishra VK et al [15].
.

GENE SYMBOL	GENE ID	PCC
CDCA3	ENSG00000111665.11	0.89
NCAPD2	ENSG00000010292.12	0.85
RAD51AP1	ENSG00000111247.14	0.83
LTBR	ENSG00000111321.10	0.75
CCNB2	ENSG00000157456.7	0.74
BIRC5	ENSG00000089685.14	0.74
CCNF	ENSG00000162063.12	0.74
TPX2	ENSG00000088325.15	0.74
PLK1	ENSG00000166851.14	0.73
PHB2	ENSG00000215021.8	0.73
UBE2C	ENSG00000175063.16	0.73
RP3-461F17.3	ENSG00000268439.5	0.73
ORC6	ENSG00000091651.8	0.73
KIF4A	ENSG00000090889.11	0.73
CDC20	ENSG00000117399.13	0.73
KIFC1	ENSG00000237649.7	0.73
KIF18B	ENSG00000186185.13	0.72
MRPL51	ENSG00000111639.7	0.72
TROAP	ENSG00000135451.12	0.72
GAPDH	ENSG00000111640.14	0.72
CENPH	ENSG00000153044.9	0.72
HMMR	ENSG00000072571.19	0.72
CCNB1	ENSG00000134057.14	0.71
MAD2L1	ENSG00000164109.13	0.71
KIF23	ENSG00000137807.13	0.71
RHNO1	ENSG00000171792.10	0.71
CDC25C	ENSG00000158402.18	0.7
TPI1	ENSG00000111669.14	0.7
NEIL3	ENSG00000109674.3	0.7
ERCC6L	ENSG00000186871.6	0.7
KIF2C	ENSG00000142945.12	0.7
SCNN1A	ENSG00000111319.12	0.7
CDCA8	ENSG00000134690.10	0.7
C17orf53	ENSG00000125319.14	0.7
EMG1	ENSG00000126749.14	0.7
NCAPH	ENSG00000121152.9	0.7
PTTG1	ENSG00000164611.12	0.7
NOP2	ENSG00000111641.10	0.69
CCNA2	ENSG00000145386.9	0.69
NDC80	ENSG00000080986.12	0.69
SKA3	ENSG00000165480.15	0.69
BLM	ENSG00000197299.10	0.69
MLF2	ENSG00000089693.10	0.69
KIF20A	ENSG00000112984.11	0.68
CENPF	ENSG00000117724.12	0.68
COPS7A	ENSG00000111652.9	0.68
ASF1B	ENSG00000105011.8	0.68
NEK2	ENSG00000117650.12	0.68
CD9	ENSG00000010278.11	0.67
CEP55	ENSG00000138180.15	0.67
KIF18A	ENSG00000121621.6	0.67
CENPU	ENSG00000151725.11	0.67
GINS1	ENSG00000101003.9	0.66
MIS18A	ENSG00000159055.3	0.66
ASPM	ENSG00000066279.16	0.66
AURKA	ENSG00000087586.17	0.66
RP5-940J5.6	ENSG00000247853.2	0.66
CDC25A	ENSG00000164045.11	0.66
PLK4	ENSG00000142731.10	0.66
GAPDHP1	ENSG00000228232.1	0.66
EZH2	ENSG00000106462.10	0.66
DIAPH3	ENSG00000139734.17	0.66
HJURP	ENSG00000123485.11	0.66
CENPA	ENSG00000115163.14	0.66
PRC1	ENSG00000198901.13	0.65
USP5	ENSG00000111667.13	0.65
TICRR	ENSG00000140534.13	0.65
TYMS	ENSG00000176890.15	0.65
CCDC77	ENSG00000120647.9	0.65
KIF15	ENSG00000163808.16	0.65
RAD51	ENSG00000051180.16	0.65
FANCI	ENSG00000140525.17	0.64
TRIP13	ENSG00000071539.13	0.64
EXO1	ENSG00000174371.16	0.64
TTK	ENSG00000112742.9	0.64
FAM72B	ENSG00000188610.12	0.64
GGH	ENSG00000137563.11	0.64
MCM2	ENSG00000073111.13	0.64
MYBL2	ENSG00000101057.15	0.64
NCAPG2	ENSG00000146918.19	0.64
SGOL1	ENSG00000129810.14	0.64
C16orf59	ENSG00000162062.14	0.64
AURKB	ENSG00000178999.12	0.64
PIF1	ENSG00000140451.12	0.64
ORC1	ENSG00000085840.12	0.64
BUB1	ENSG00000169679.14	0.63
FKBP4	ENSG00000004478.7	0.63
UBE2T	ENSG00000077152.9	0.63
XRCC2	ENSG00000196584.2	0.63
DLGAP5	ENSG00000126787.12	0.63
DEPDC1	ENSG00000024526.16	0.63
CENPN	ENSG00000166451.13	0.63
CDCA5	ENSG00000146670.9	0.63
TBC1D2	ENSG00000095383.19	0.63
GINS2	ENSG00000131153.8	0.62
CKS2	ENSG00000123975.4	0.62
E2F1	ENSG00000101412.12	0.62
AUNIP	ENSG00000127423.10	0.62
CDKN3	ENSG00000100526.19	0.62
CDT1	ENSG00000167513.8	0.61

FUNCTIONS

- DNA repair/DNA damage
- Differentiation
- Metabolism
- FOXM1 deubiquitinase

Supplementary Table S7. Critical FOXM1 role in pancreatic cancer patients.
GEPIA generated a list of top 100 genes that have similar expression pattern ranked by Pearson correlation coefficient (PCC) in PAAD TCGA dataset. In yellow genes involved in DNA damage and repair, in green genes involved in differentiation pathways, in blue genes involved in metabolism process and in red USP5, the deubiquitinase gene involved in FOXM1 deubiquitination.

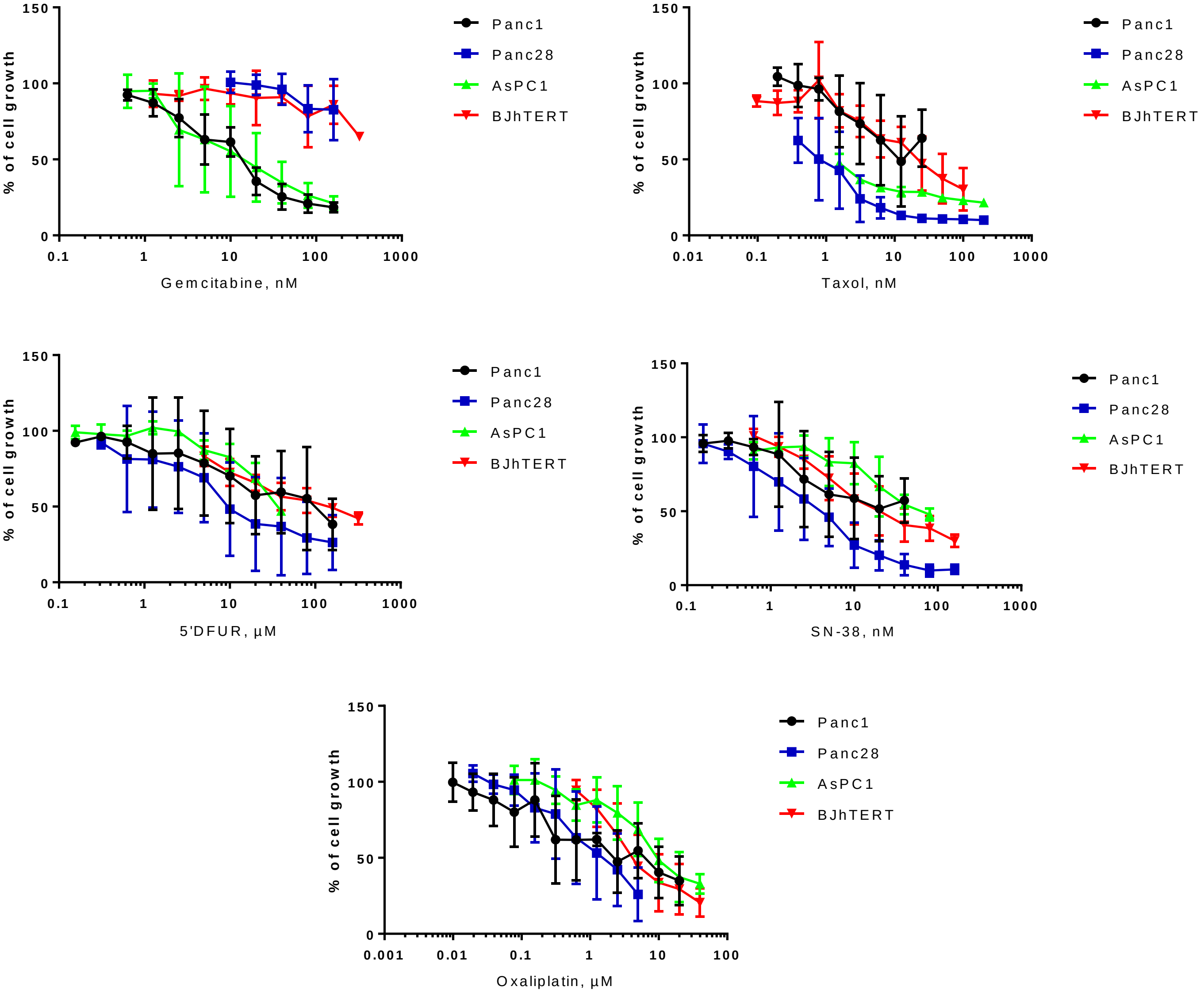

Supplementary Figure S1. **Chemotherapy effect on pancreatic cancer cells.**
Cytotoxicity curves for gemcitabine, taxol, 5'DFUR, SN-38 and oxaliplatin. ASPC1, PANC1, PANC28 and BJhTERT cells were treated for 96h with increasing concentration of single drugs (doses are reported in the figure). Cell growth expressed as percentage of control was assessed by sulforhodamine B colorimetric assay.

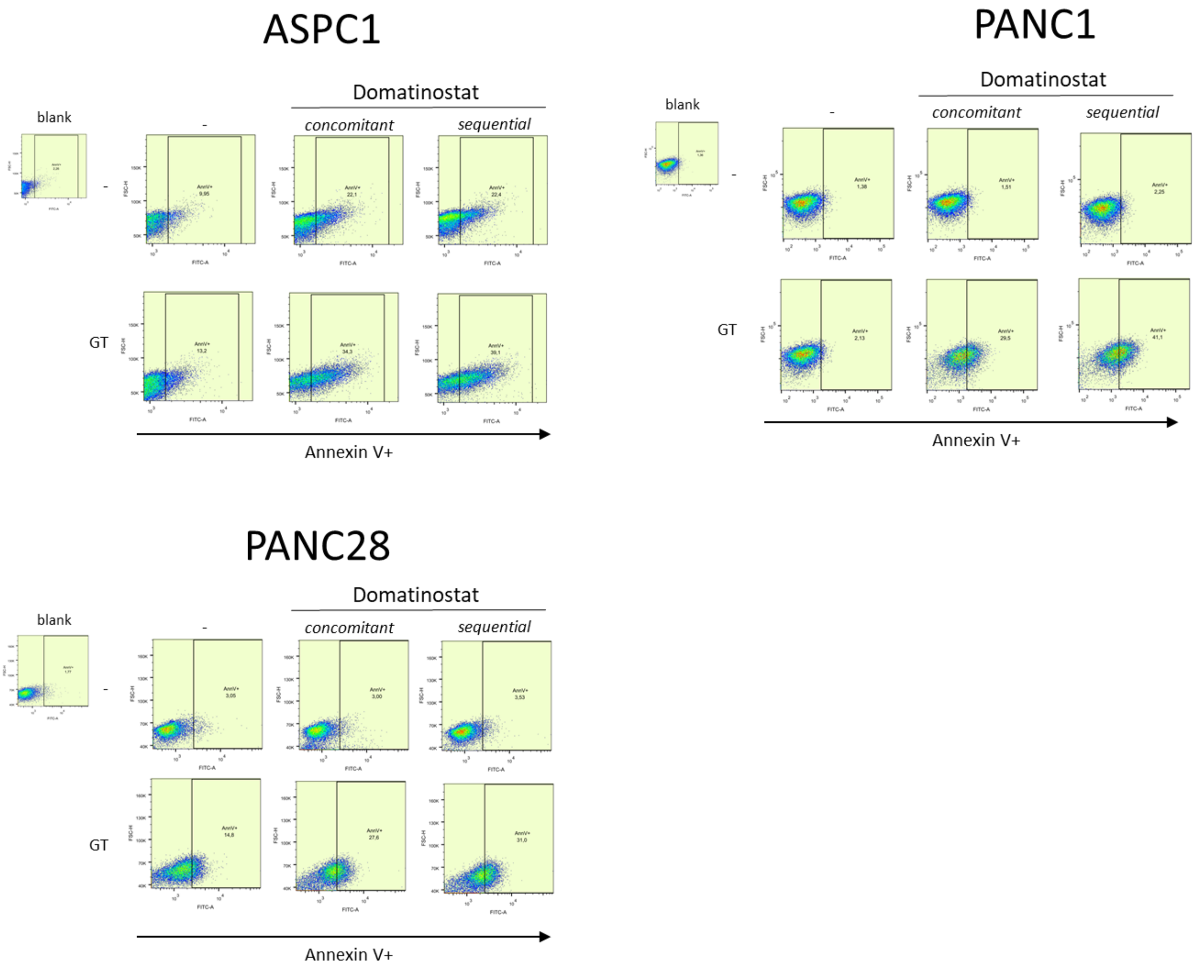

Supplementary Figure S2. **Domatinostat plus gemcitabine/taxol combination induces Annexin-V exposure.**

Flow cytometry analysis of Annexin-V shows the outcome of the induced chemosensitivity in PANC1, PANC28, ASPC1 cells when domatinostat (0.5 μM) is combined with gemcitabine/taxol (GT) (IC50 at 96h) for 24h and when domatinostat (0.5 μM) is given 24h before gemcitabine/taxol (GT) (IC50 at 96h) for 24h.

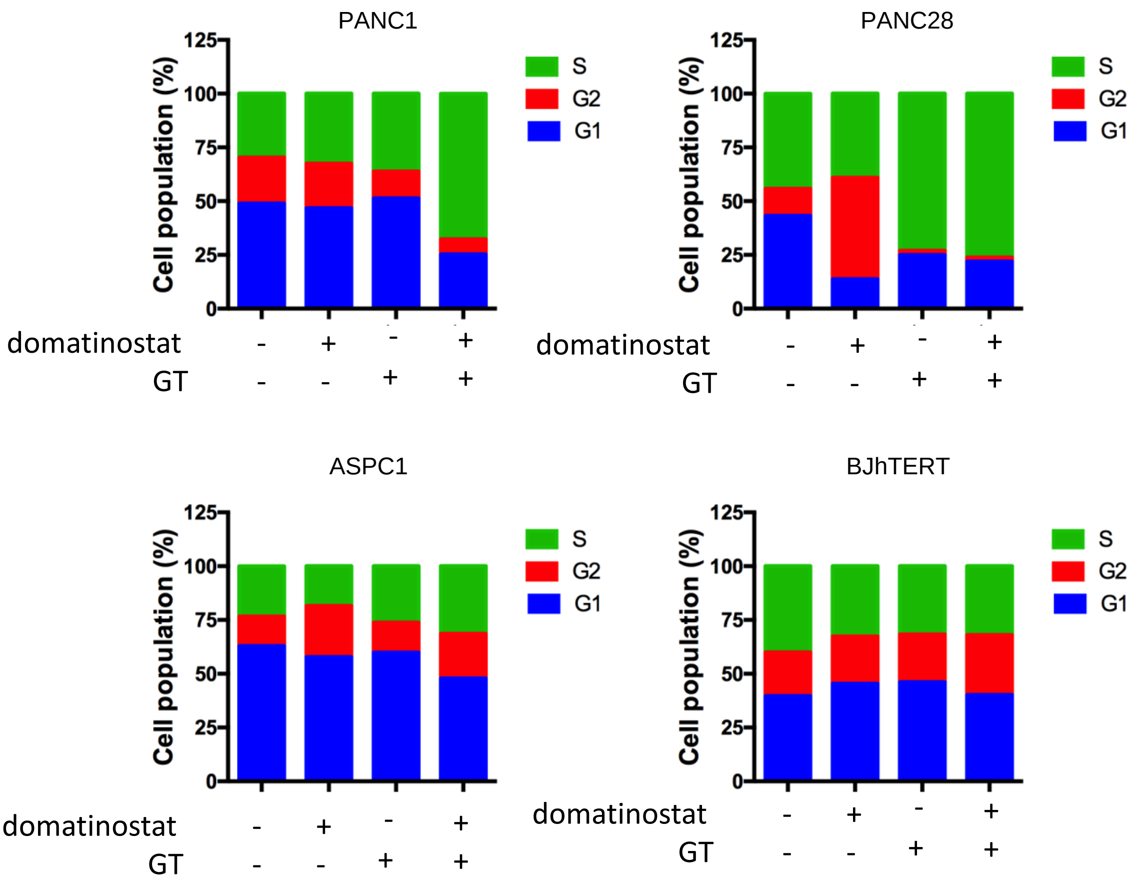

Supplementary Figure S3. D**omatinostat plus gemcitabine/taxol combination induces cell cycle perturbation and a clear block in phase S.**

Cell cycle analysis was performed in PANC1, PANC28, ASPC1 and BJhTERT cell lines. The percentage of G1, S and G2/M population were analyzed by flow cytometry on cells treated for 48 h with or without domatinostat 0.5 µM and gemcitabine/taxol (IC50 measured for each cell lines) for 48 hours.

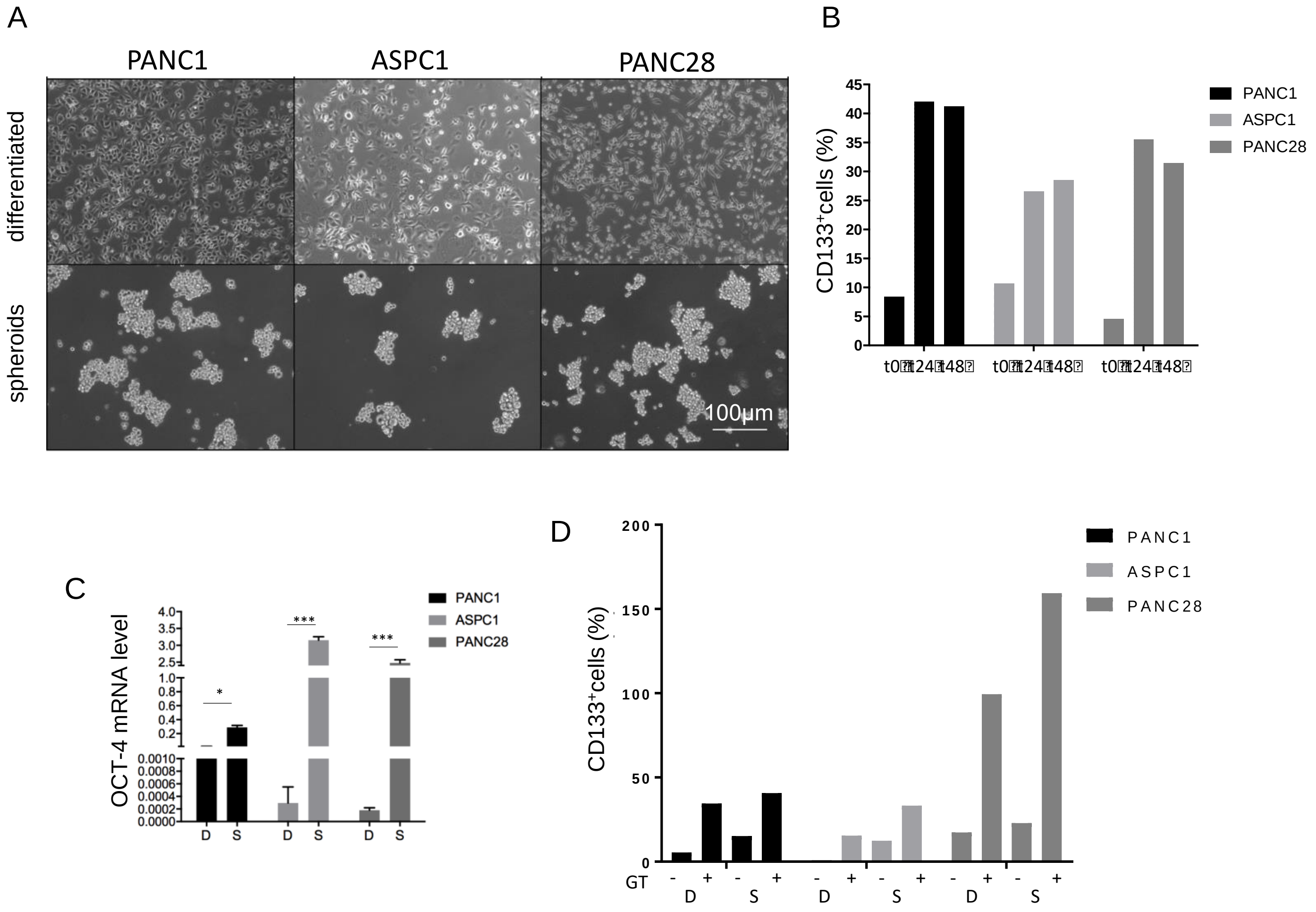

Supplementary Figure 4. Pancreatic cancer cells enriched in stem-cell features, culturing model validation.

A. Images of PANC1, PANC28, ASPC1 cells cultured in standard condition are referred as *differentiated (D)* and PANC1, PANC28, ASPC1 cells cultured in sphere medium (see Material and Methods) in low-adherent wells for 48h are referred as *spheroids (S)*. White bar 100µM. Magnification 10X. **B.** Flow cytometry shows increased levels of associated stem-cell marker CD133 in PANC1, PANC28, ASPC1 culturing as spheroids for 24 and 48 hours. **C.** qRT-PCR analysis shows OCT-4 levels increase in PANC1, PANC28 and ASPC1 spheroids (S) compared to PANC1, PANC28 and ASPC1 differentiated cells (D). **D.** Flow cytometry shows increased levels of associated stem-cell marker CD133 more in spheroids than differentiated cells upon gemcitabine/taxol (IC50 at 96h) 24 hours treatment.

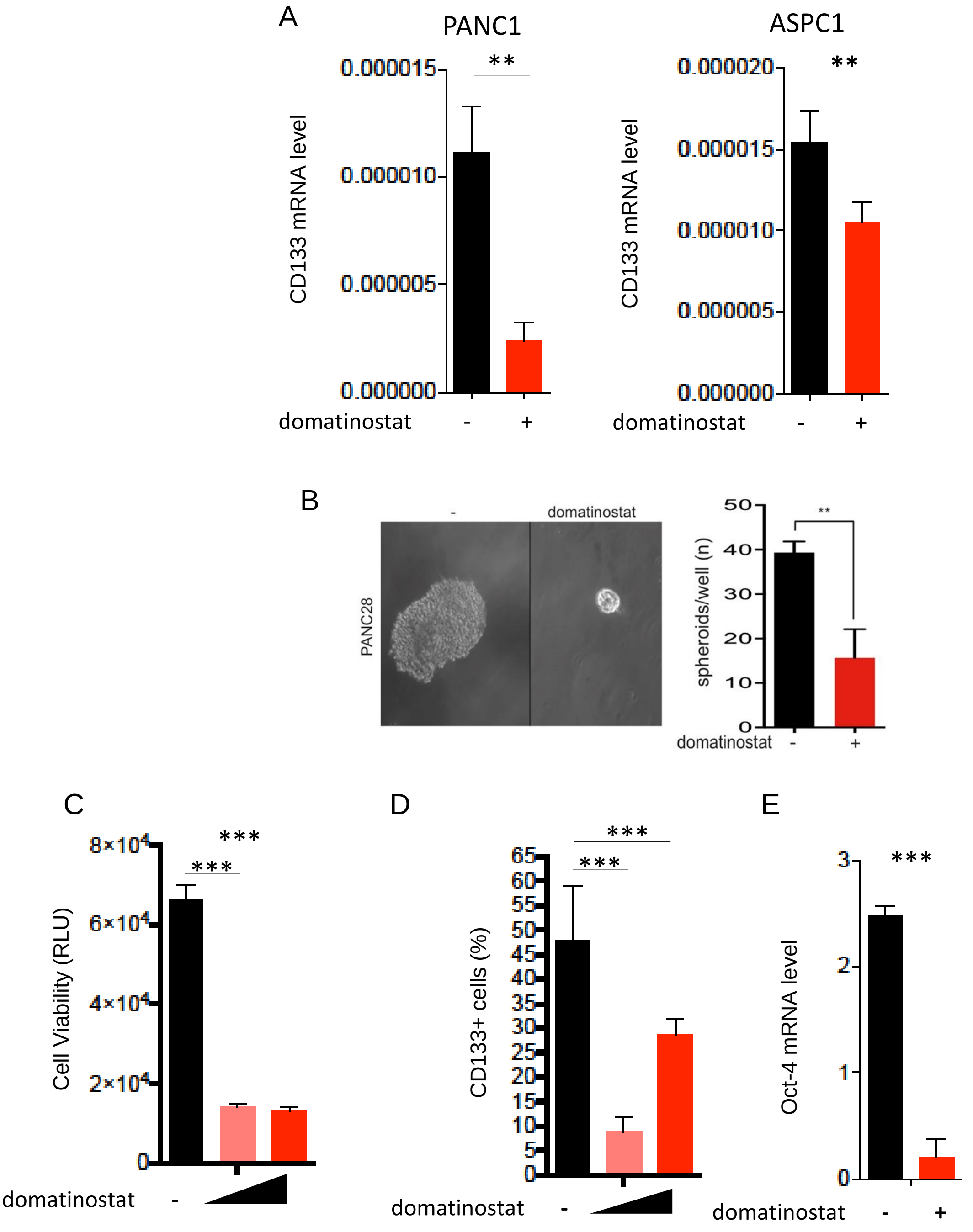

Supplementary Figure 5. Domatinostat effects on CSC population.

A qRT-PCR analysis shows CD133 levels drop when PANC1 and ASPC1 spheroids are treated with domatinostat (0.5 µM) for 16h. **B.** The effect of domatinostat (0.5 µM) on PANC28 spheroid cultures. Cells seeded as described in methods were collected 7 days after treatment. A representative image of one spheroid for each condition is shown (white scale bar: 50 µm, magnification 20X). On the right, bar graphs show the numbers of spheroids for well (mean±SD of 2 or more separate experiments each one with technical triplicate). **C.** PANC28 spheroids viability treated with and without domatinostat (0.5 µM and 1 µM) was assessed by cell titer luminescence assay (mean±SD of 2 or more separate experiments each one with technical triplicate). **D.** Flow cytometry shows decreased levels of associated stem cell marker CD133 upon increasing concentration of domatinostat (0.5 and 1 µM) for 16h. **E.** qRT-PCR analysis shows Oct-4 levels drop when spheroids are treated with domatinostat (0.5 µM) for 16h. **F.** qRT-PCR analysis shows CD133 levels drop when PANC28 spheroids are treated with domatinostat (0.5 µM) for 8h.

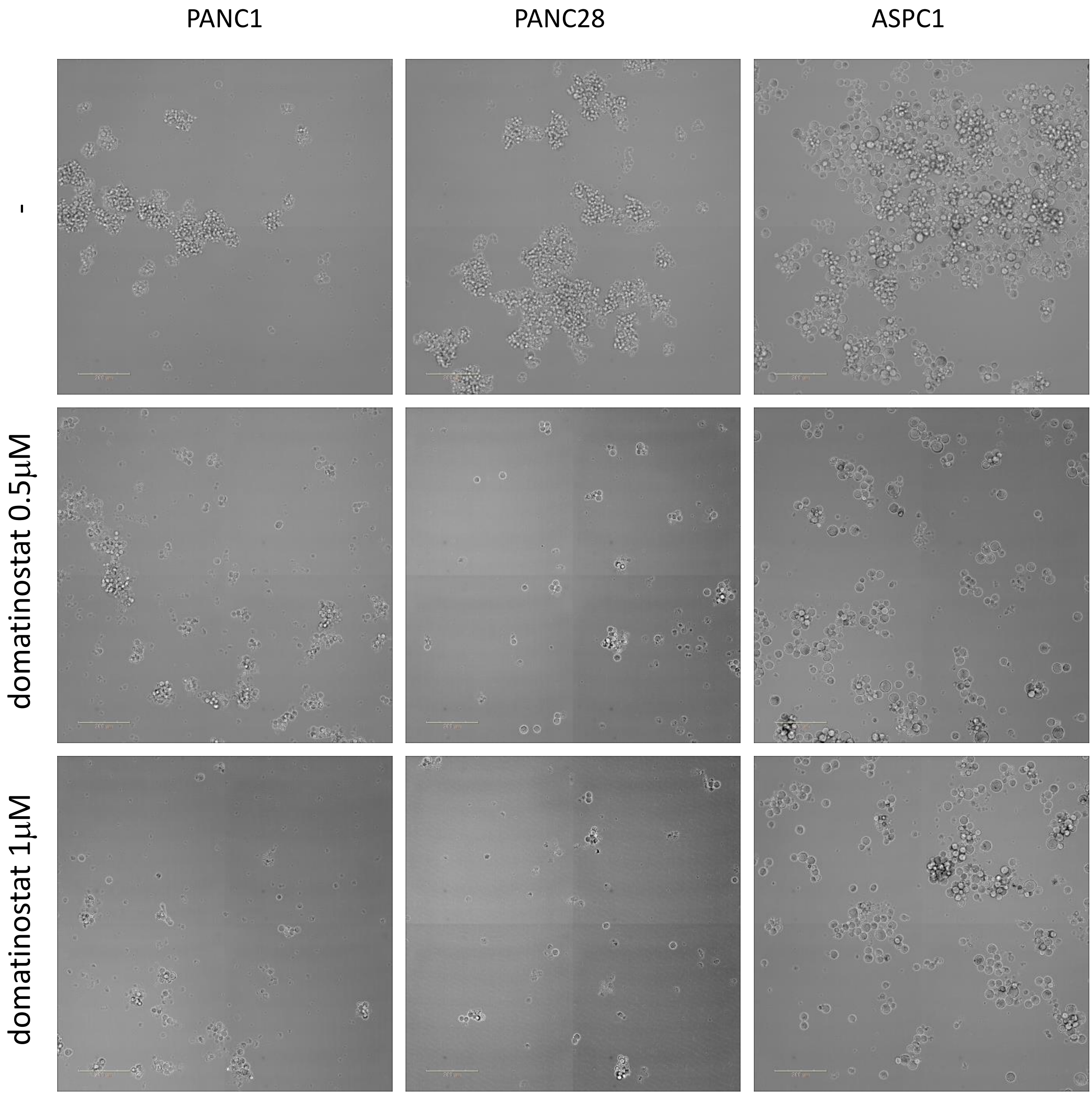

Supplementary Figure S6. The effect of domatinostat on PDAC spheroid cultures.
PANC1, PANC28 and ASPC1 cells seeded in sphere medium were treated with and without domatinostat (0.5 μM and 1 μM) and collected 3 days after treatment. Images of spheroids for each condition in a representative experiment are by Opera Phenix confocal microscopy (magnification 20X-white scale bar: 200μm).

A

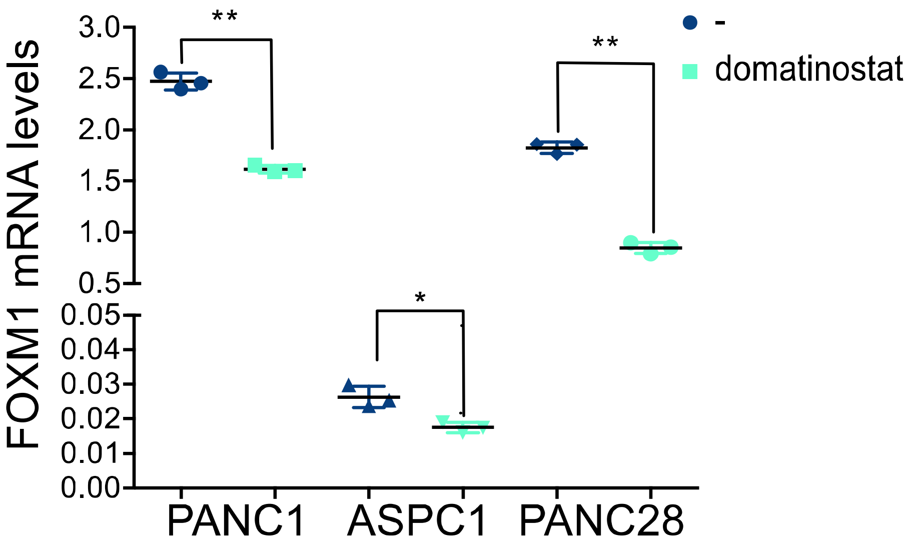

B

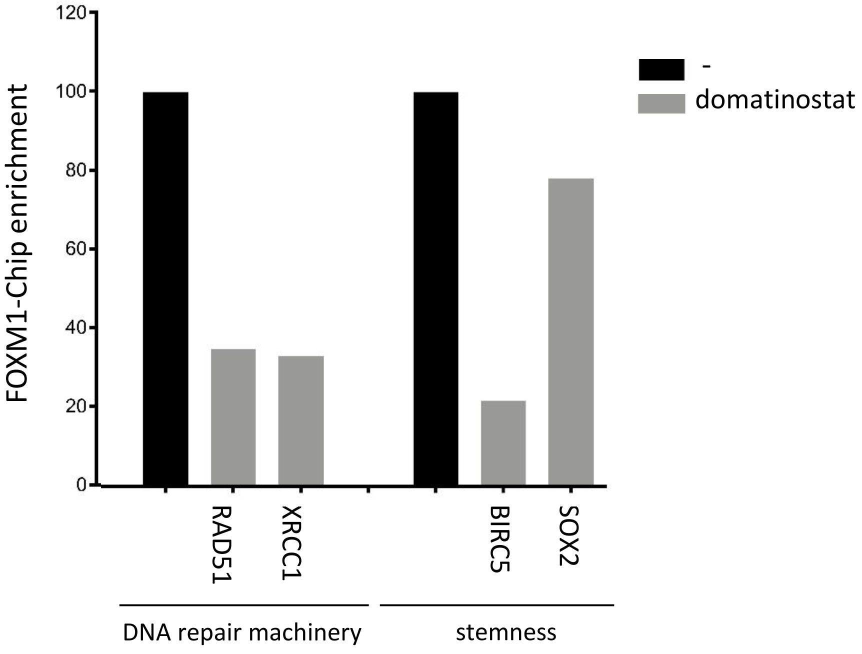

Supplementary Figure S7. Domatinostat effects on FOXM1 expression.
A. qRT-PCR analysis shows FOXM1 levels drop when PANC1, PANC28 and ASPC1 spheroids are treated with domatinostat (0.5 μM) for 16h. **B.** ChIP-qPCR analysis showing the relative decrease of enrichment. Data obtained on immunoprecipitated fractions were normalized to input chromatin (IP/Input). After 16 hours of domatinostat (1μM) treatment, PANC1 spheroids are crosslinked and a chromatin immunoprecipitation has been performed as described in Materials and Methods. A representative single experiment is reported.

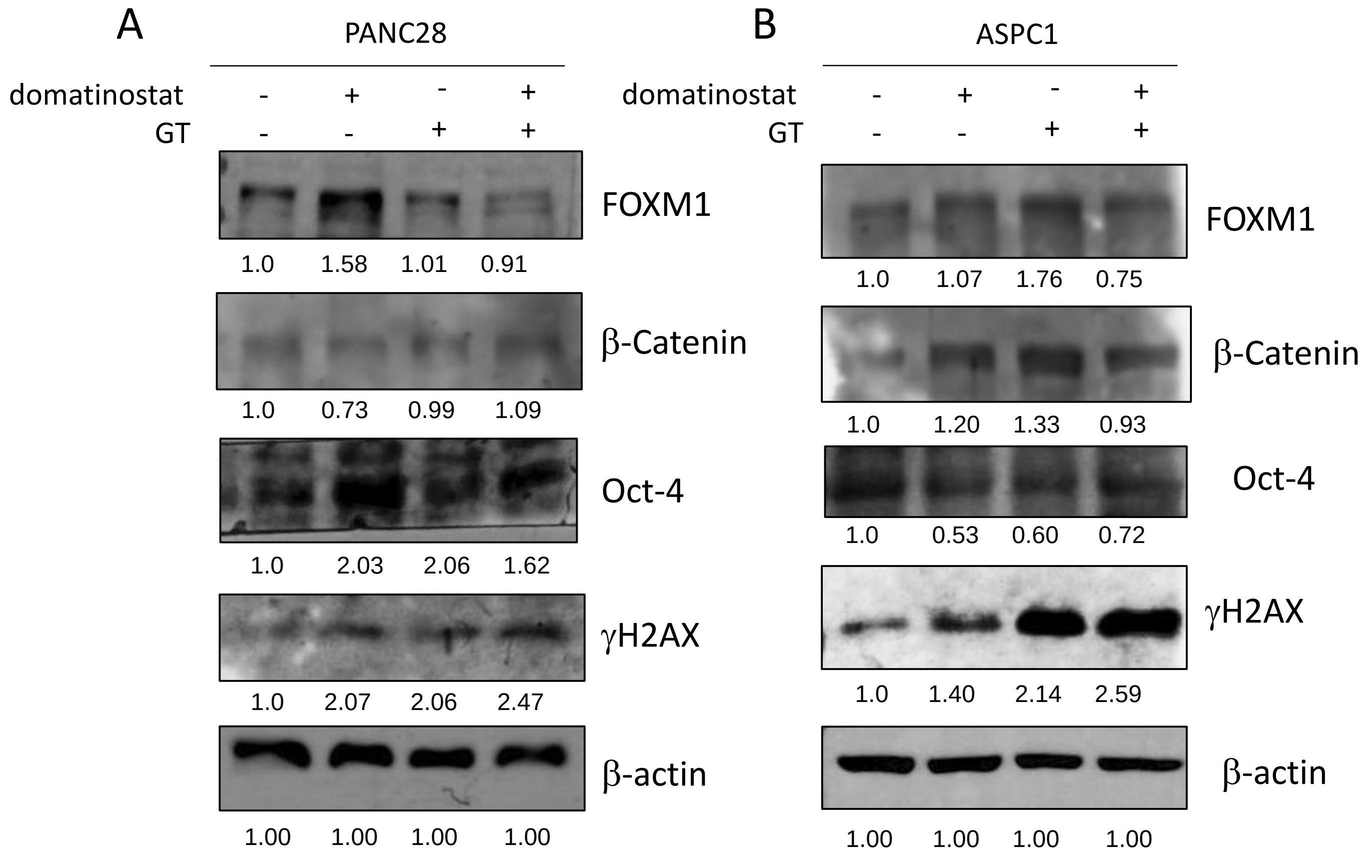

Supplementary Figure S8. **Domatinostat modulates FOXM1 activity**
Western Blot analysis shows decreased protein levels of FOXM1 and associated stem-cell markers (β-Catenin and Oct-4) and increased γH2AX protein as stress marker in PANC28 **(A)** and in ASPC1 spheroids **(B)**, treated for 16 hours with domatinostat, GT (IC50 at 96h) and their combination. β-actin serves as control for equal protein loading.

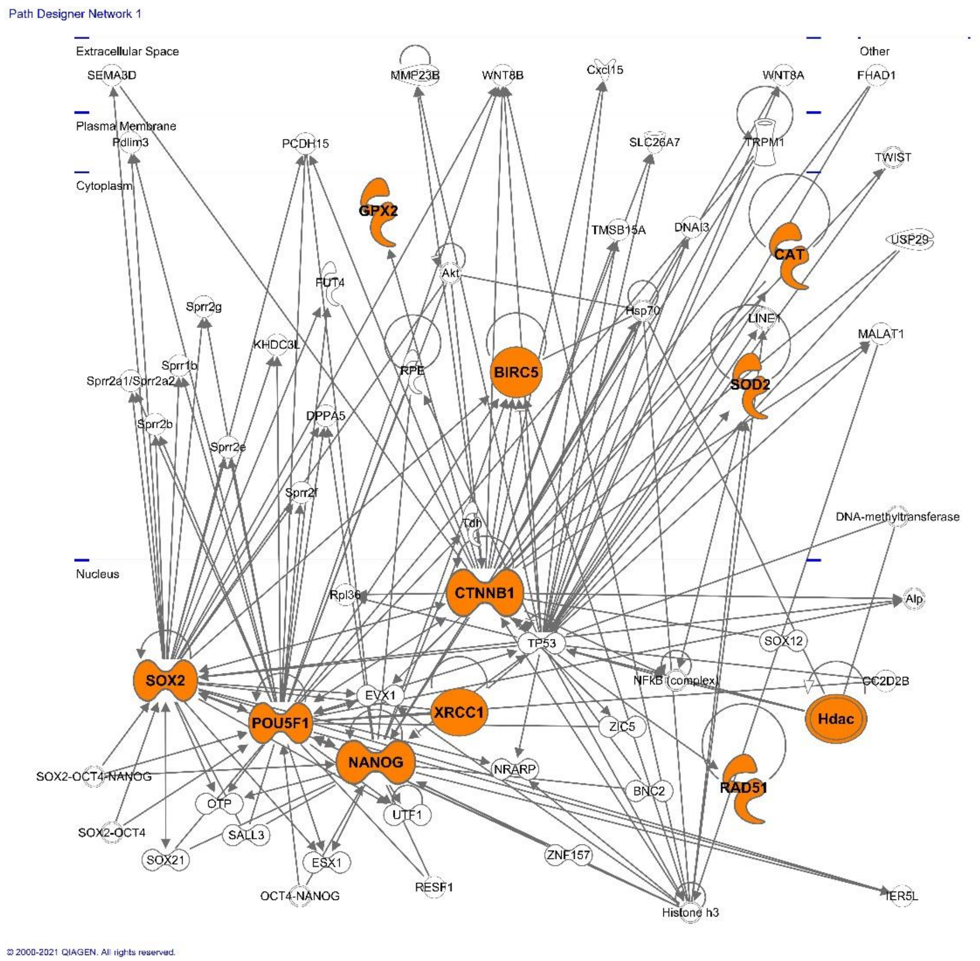

Upstream Regulator	Molecule Type	p-value of overlap	Target Molecules in Dataset
FOXM1	transcription regulator	4,20E-16	BIRC5,CAT,CTNNB1,NANOG,POU5F1,SOD2,SOX2,XRCC1
SOX17	transcription regulator	1,10E-09	CTNNB1,NANOG,POU5F1,SOX2
PRMT5	enzyme	7,63E-09	BIRC5,NANOG,POU5F1,SOX2
SIRT1	transcription regulator	1,53E-08	BIRC5,CAT,CTNNB1,NANOG,POU5F1,SOD2
FOXD3	transcription regulator	1,91E-08	NANOG,POU5F1,SOX2
TBX3	transcription regulator	3,94E-08	NANOG,POU5F1,SOX2
E2F1	transcription regulator	5,27E-08	BIRC5,CTNNB1,NANOG,RAD51,SOD2,XRCC1
GMNN	transcription regulator	5,57E-08	CTNNB1,NANOG,POU5F1,SOX2
EPAS1	transcription regulator	6,57E-08	CAT,NANOG,POU5F1,SOD2,SOX2
YY2	transcription regulator	7,06E-08	NANOG,POU5F1,SOX2

© 2000-2021 QIAGEN. All rights reserved.

Supplementary Figure S9. **A network analysis revealed FOXM1 as upstream regulator of significant domatinostat-modulated proteins involved in stemness, oxidative stress and DNA repair.**
FOXM1 came out in an Ingenuity Pathway analysis as main significant upstream regulator in a pathway that directly links all domatinostat-targets. Specifically, we used as input to generate the pathway: stemness proteins (BIRC5, NANOG, POU5F1 and SOX2), oxidative stress and DNA damage-related proteins (such as XRCC1, RAD51, SOD2, CAT and GPX2) and HDAC protein.

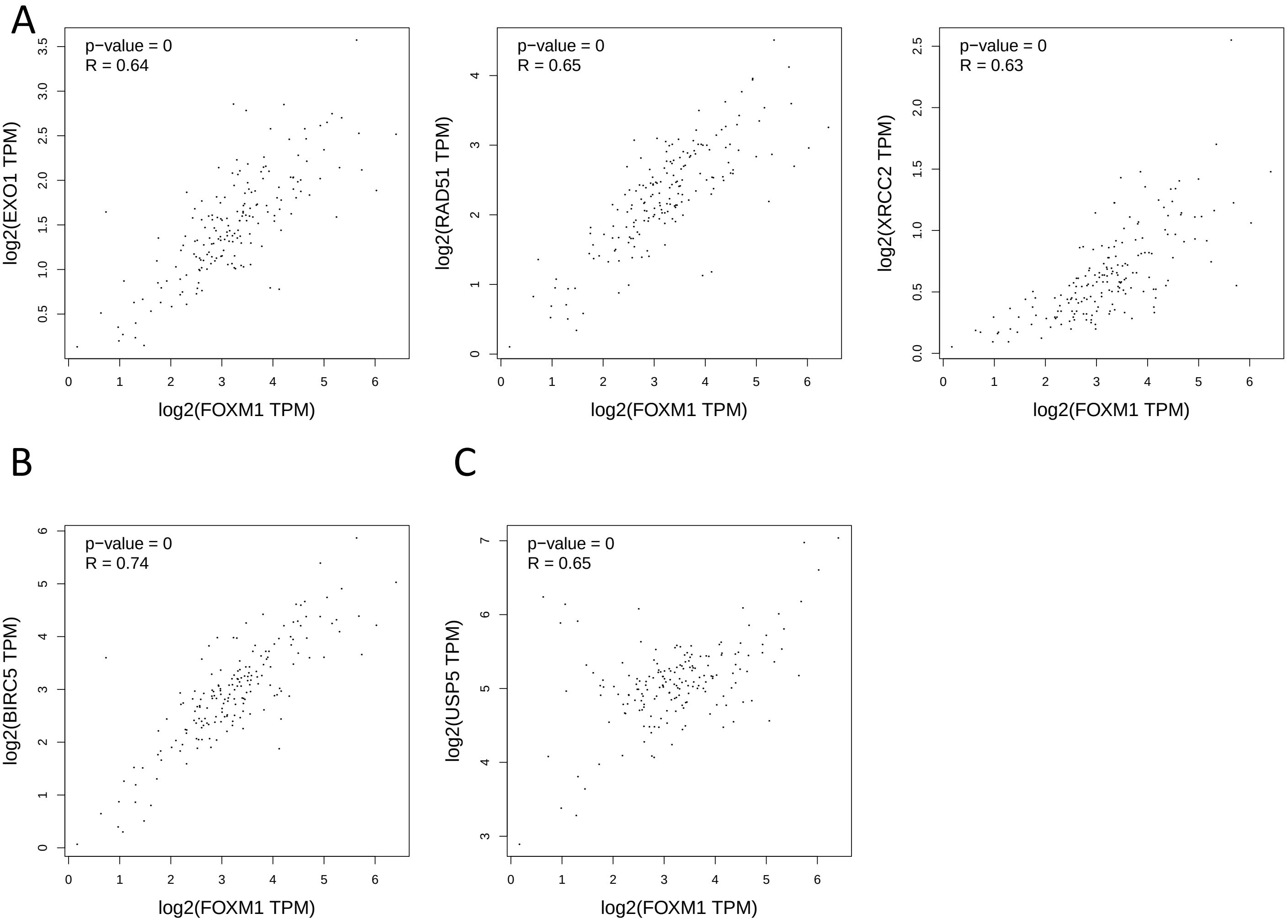

Supplementary Figure S10. **Critical FOXM1 role in pancreatic cancer patients.**

A. Scatter plots of mRNA levels (RSEM normalized values) of FOXM1 and representative DNA-damage induced genes (EXO1, RAD51, XRCC2,) in the TCGA PAAD cohort. **B.** Scatter plots of mRNA levels (RSEM normalized values) of FOXM1 and BIRC5 in the TCGA PAAD cohort. **C.** Scatter plots of mRNA levels (RSEM normalized values) of FOXM1 and USP5 in the TCGA PAAD cohort.

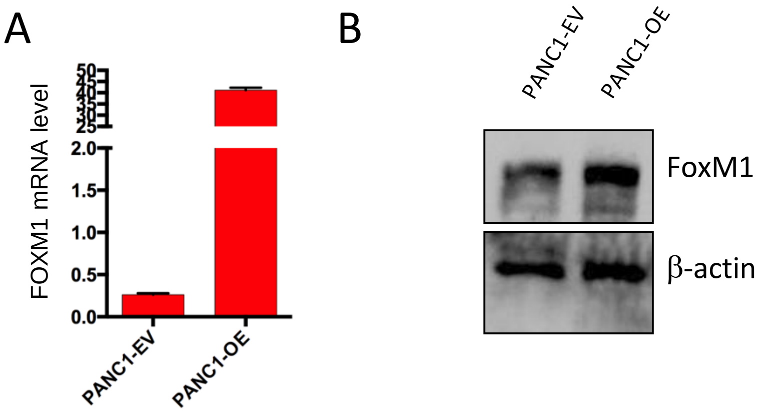

Supplementary Figure S11. **domatinostat modulates FOXM1 activity**
The expression of FOXM1 in PANC1 cells transfected with FOXM1(OE-FOXM1) or with empty vector (EV-FOMX1) evaluated by transcript quantification **(A)** or by WB analysis, β-actin serves as control for equal protein loading **(B)**.

A

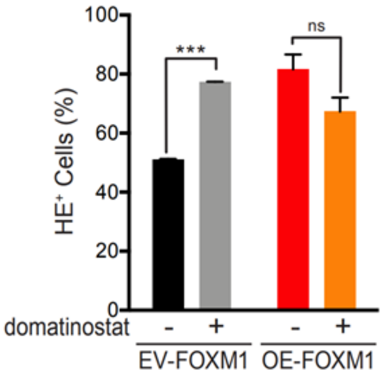

B

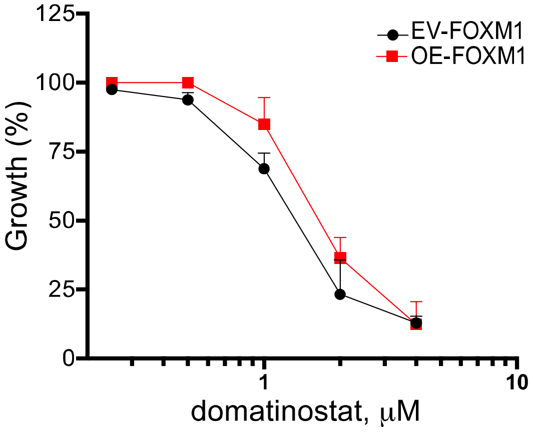

Supplementary Figure S12. **Domatinostat modulates FOXM1 activity**

A. Cellular ROS in OE-FOXM1 and EV-FOXM1 PANC1, treated or untreated with domatinostat (0.5μM) for 16h, visualized by Hydroethidine staining. **B.** PANC1 cells transfected with FOXM1 (OE-FOXM1) or with empty vector (EV-FOMX1) were treated for 96h with increasing concentration of domatinostat. Cell growth expressed as percentage of control was assessed by sulforhodamine B colorimetric assay.

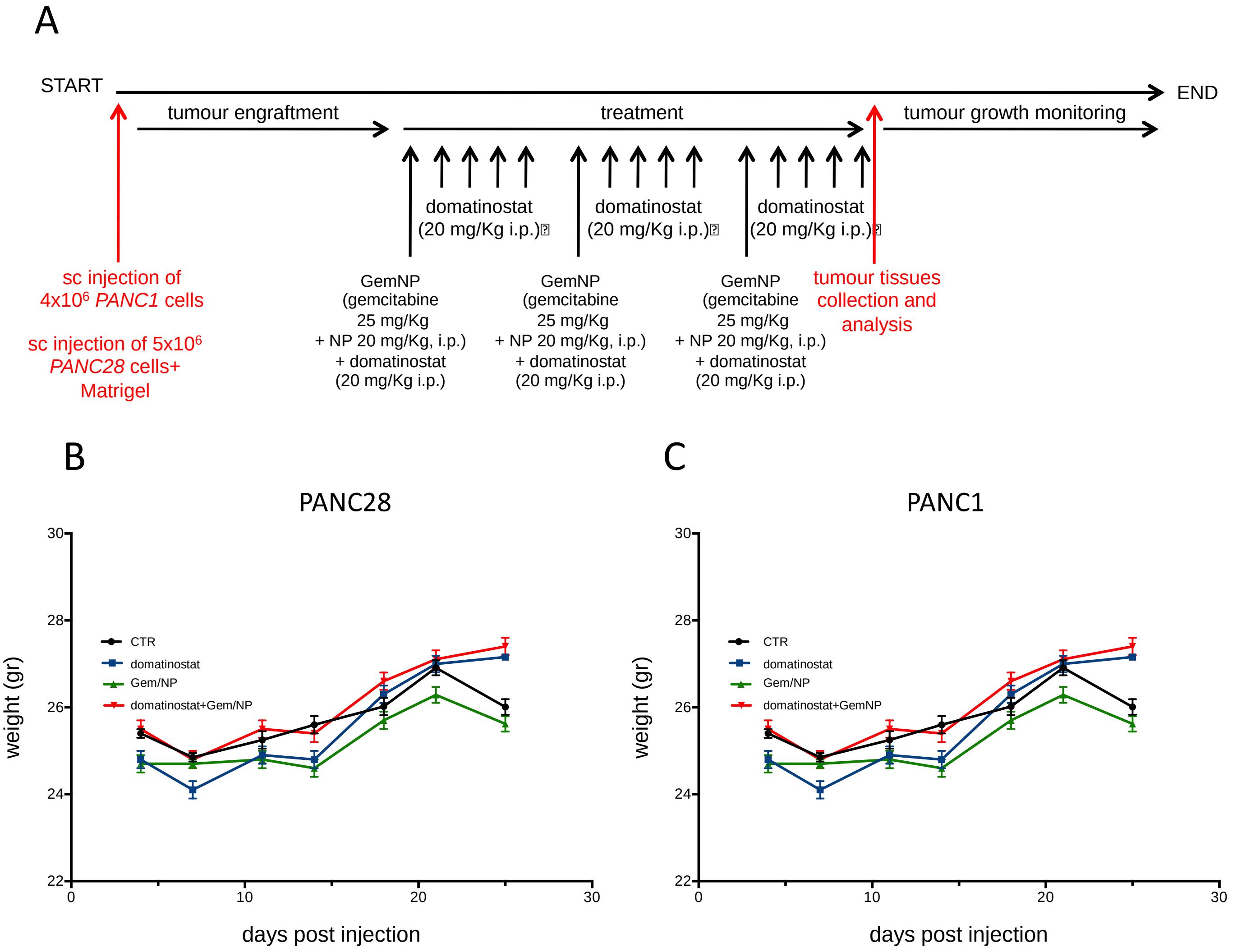

Supplementary Figure S13. **Domatinostat does not exert toxic effect alone and in combination *in vivo*.**

A. Representative schedule of in vivo treatments with timing and agents concentration **B-C.** Mice body weight measured two times/week in PANC28 and PANC1 xenografts.

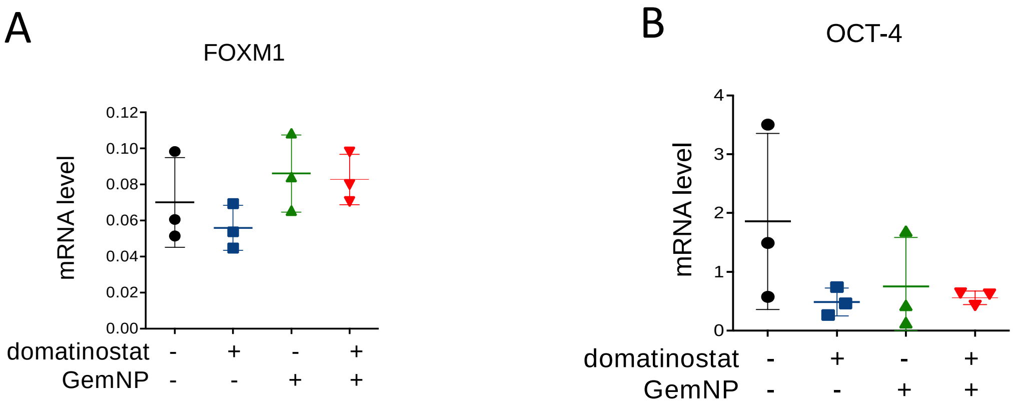

Supplementary Figure S14. **Domatinostat synergistically improves overall survival of PANC1 mice affecting FOXM1 and OCT4 expression.**

A. FOXM1 mRNA expression determined by qRT-PCR in PANC1 samples obtained from three PANC1 xenografts from each treatment group at the end of treatment. **B.** OCT-4 mRNA expression determined by qRT-PCR in PANC1 samples obtained from three PANC1 xenografts from each treatment group at the end of treatment. β-actin was used as housekeeping control gene.
